# Exposure to PFAS chemicals induces sex-dependent alterations in key rate-limiting steps of lipid metabolism in liver steatosis

**DOI:** 10.3389/ftox.2024.1390196

**Published:** 2024-06-05

**Authors:** Archana Hari, Mohamed Diwan M. AbdulHameed, Michele R. Balik-Meisner, Deepak Mav, Dhiral P. Phadke, Elizabeth H. Scholl, Ruchir R. Shah, Warren Casey, Scott S. Auerbach, Anders Wallqvist, Venkat R. Pannala

**Affiliations:** ^1^ Department of Defense Biotechnology High Performance Computing Software Applications Institute, Telemedicine and Advanced Technology Research Center, U.S. Army Medical Research and Development Command, Fort Detrick, MD, United States; ^2^ The Henry M. Jackson Foundation for the Advancement of Military Medicine, Inc., Bethesda, MD, United States; ^3^ Sciome LLC, Research Triangle Park, NC, United States; ^4^ Division of Translational Toxicology, National Institute of Environmental Health Sciences, Research Triangle Park, NC, United States

**Keywords:** PFAS, PAH, steatosis, MIE, PPAR signaling, bile acid synthesis, gluconeogenesis, lipid synthesis

## Abstract

Toxicants with the potential to bioaccumulate in humans and animals have long been a cause for concern, particularly due to their association with multiple diseases and organ injuries. Per- and polyfluoro alkyl substances (PFAS) and polycyclic aromatic hydrocarbons (PAH) are two such classes of chemicals that bioaccumulate and have been associated with steatosis in the liver. Although PFAS and PAH are classified as chemicals of concern, their molecular mechanisms of toxicity remain to be explored in detail. In this study, we aimed to identify potential mechanisms by which an acute exposure to PFAS and PAH chemicals can induce lipid accumulation and whether the responses depend on chemical class, dose, and sex. To this end, we analyzed mechanisms beginning with the binding of the chemical to a molecular initiating event (MIE) and the consequent transcriptomic alterations. We collated potential MIEs using predictions from our previously developed ToxProfiler tool and from published steatosis adverse outcome pathways. Most of the MIEs are transcription factors, and we collected their target genes by mining the TRRUST database. To analyze the effects of PFAS and PAH on the steatosis mechanisms, we performed a computational MIE-target gene analysis on high-throughput transcriptomic measurements of liver tissue from male and female rats exposed to either a PFAS or PAH. The results showed peroxisome proliferator-activated receptor (PPAR)-α targets to be the most dysregulated, with most of the genes being upregulated. Furthermore, PFAS exposure disrupted several lipid metabolism genes, including upregulation of fatty acid oxidation genes (*Acadm*, *Acox1*, *Cpt2*, *Cyp4a1*-*3*) and downregulation of lipid transport genes (*Apoa1*, *Apoa5*, *Pltp*). We also identified multiple genes with sex-specific behavior. Notably, the rate-limiting genes of gluconeogenesis (*Pck1*) and bile acid synthesis (*Cyp7a1*) were specifically downregulated in male rats compared to female rats, while the rate-limiting gene of lipid synthesis (*Scd*) showed a PFAS-specific upregulation. The results suggest that the PPAR signaling pathway plays a major role in PFAS-induced lipid accumulation in rats. Together, these results show that PFAS exposure induces a sex-specific multi-factorial mechanism involving rate-limiting genes of gluconeogenesis and bile acid synthesis that could lead to activation of an adverse outcome pathway for steatosis.

## 1 Introduction

Per- and polyfluoro alkyl substances (PFAS) are synthetic compounds, comprised of at least one aliphatic unit of perfluorocarbon, with surfactant and heat-resistant properties that make them useful for many commercial products (AbdulHameed et al., 2019; Roth et al., 2021; Wang et al., 2022; Louisse et al., 2023). However, despite their benefits, exposure to PFAS chemicals has been associated with an increased occurrence of adverse health outcomes (Khalil et al., 2016; Fenton et al., 2021; Schultz et al., 2023) due to their bioaccumulation, with PFAS chain length and chemical functional group attachment affecting the bioaccumulation process (Fenton et al., 2021; Haug et al., 2023). In addition, the PFAS dose and length of exposure have been shown to affect the adverse outcomes (Wan et al., 2012; Fenton et al., 2021). Long-chain PFAS [“legacy” PFAS, such as perfluorooctanoic acid (PFOA)] have longer half-lives and consequently more detrimental outcomes than shorter-chain PFAS [“emerging” PFAS, such as ammonium 2,3,3,3-tetrafluoro-2-(heptafluoropropoxy)-propanoate (HFPO-DA)], leading to the discontinuation of long-chain PFAS production and use of shorter-chain PFAS as safer alternatives (Heintz et al., 2022). The United States Environmental Protection Agency (EPA) has announced a National Primary Drinking Water Regulation (NPDWR) to establish legally enforceable levels, called Maximum Contaminant Levels (MCLs), and health-based, non-enforceable Maximum Contaminant Level Goals (MCLGs) for six PFAS in drinking water (PFOA and PFOS as individual contaminants and PFHxS, PFNA, PFBS, and HFPO-DA as a PFAS mixture). The regulation requires monitoring the levels of the six previously mentioned PFAS in public water systems and implementation of methods to reduce contamination if initial monitoring reveals levels exceeding the MCLs (United States Environmental Protection Agency, 2024a; United States Environmental Protection Agency, 2024b). Due to such increasing concerns about the persistent bioaccumulation and toxic associations of PFAS chemicals, particularly in the liver, there is a growing need to study the effects of PFAS compounds at various exposure lengths and doses as well as during acute exposures.

Similarly, polycyclic aromatic hydrocarbons (PAH), another class of chemical made of fused carbon and hydrogen aromatic rings that are byproducts of the incomplete combustion of organic compounds, are known to bioaccumulate in marine organisms (D’Adamo et al., 1997; Liu et al., 2023). In addition, long-term PAH exposure has been associated with tumor development in various organs in humans (Rengarajan et al., 2015; Mallah et al., 2022). Similar to PFAS toxicants, the dose and duration of PAH exposure are known to affect the severity of the outcome (Patel et al., 2020). While the carcinogenic effects of PAH make them a chemical of concern, there is limited information on the toxic effects of PAH on the liver, which is a primary site of PAH metabolization (Zhou et al., 2024). Current studies only report associations of PAH exposure with impaired liver function (Xu et al., 2021; Choi et al., 2023; Zhou et al., 2024) and lack information on the molecular mechanisms underlying specific hepatotoxic endpoints.

Most of the existing studies on PFAS and PAH exposure include only males, and the few studies that include both males and females provide conflicting evidence regarding the sex-dependent effects of PFAS. Based on their experiments on mice, Roth et al. reported that female mice show a greater likelihood of developing hepatic toxicity due to PFAS exposure (Roth et al., 2021). Based on their analysis of data from male and female patients and murine data from Schlezinger et al. (Schlezinger et al., 2020), Sen et al. reported that females are likely more sensitive to PFAS exposure than males (Sen et al., 2022). In contrast, Kim et al. reported that exposure to perfluorooctane sulfonate (PFOS) caused a larger disruption of lipid metabolism in the liver of male rats compared to female rats and that the no-observed-adverse-effect level (NOAEL) value was lower in male *versus* female rats (Kim et al., 2011). The discrepancy observed in these results can be due to variation in study design, such as choice of chemical, animal species and diet, and length and means of toxicant exposure, which makes it further challenging to compare such studies directly and make conclusions regarding sex-specific effects of the toxicants. This demands inferences from experiments that follow the same protocol to study various chemicals and both sexes. Furthermore, most epidemiological studies on human populations indirectly assessed PFAS toxicity by measuring serological biomarkers of injury, such as alanine aminotransferase (ALT) and aspartate aminotransferase (AST). For example, Costello et al. (Costello et al., 2022) provided a detailed systematic review of evidence from studies that associated PFAS exposure and ALT levels, in humans and rodents, and concluded that PFAS exposure correlated with increased ALT levels, indicating liver injury. Similarly, various human-health assessment studies have associated PFAS exposure with alterations in serum lipids and increased ALT (Steenland et al., 2009; Nelson et al., 2010; Gallo et al., 2012; Eriksen et al., 2013; Attanasio, 2019; Li et al., 2020). Some studies even associated PFAS exposure with sex-dependent outcomes, such as thyroid dysfunction and reproductive disorders (Melzer et al., 2010; Blake et al., 2018; Hammarstrand et al., 2021; Xie et al., 2021; Sang et al., 2024). Studies using serum measurements of liver injury markers, however, do not provide insights into the PFAS-induced molecular mechanisms of liver injury, particularly for specific endpoints, such as steatosis, hepatocellular carcinoma, and inflammation.

Of all the possible toxicity-induced liver injury phenotypes, many rodent and human studies have consistently linked PFAS exposure with various degrees of lipid accumulation in the liver (Wan et al., 2012; Bagley et al., 2017). The increased buildup of lipids in the liver leads to an adverse outcome called hepatic steatosis, which can progress to liver cirrhosis or non-alcoholic fatty liver disease (NAFLD). Thus, understanding the progression from exposure to steatosis can help elucidate the molecular mechanisms of PFAS-induced liver injury via steatosis. Steatosis adverse outcome pathways (AOPs) are frameworks that link a molecular initiating event (MIE) (such as molecular binding to a toxicant) to steatosis through a sequence of biological steps organized into intermediate effects (e.g., upregulation of specific genes or increased fatty acid synthesis) and key events (such as liver triglyceride accumulation) (Ankley et al., 2010; Mellor et al., 2016). Although researchers have described various steatosis AOPs, how PFAS exposure leads to steatosis via the previously described AOPs remains underexplored to our knowledge.

While existing studies report various metabolic pathways and mechanisms by which PFAS cause steatosis, they do not describe the effect of dose response or organism sex and include an analysis of only a limited set of pathways. Thus, there is a need for further studies on the effects of PFAS and PAH exposure on the liver that explore known AOP mechanisms of steatosis induction and how the mechanisms vary by sex.

In this study, we analyzed rat gene expression responses to PFAS and PAH with regard to steatosis AOP mechanisms, with a focus on identifying responses that are dependent on toxicant class, dose, or sex. We obtained gene expression data, which are publicly available in the National Toxicology Program (NTP) Chemical Effects in Biological Systems databases, from previously published reports (Auerbach et al., 2023a; Auerbach et al., 2023b; Auerbach et al., 2023c; Auerbach et al., 2023d) that detailed experiments where male and female Sprague Dawley rats were subjected to a daily dose of either PFAS or PAH for five consecutive days and transcriptomics data were collected on the sixth day for targeted RNA-sequencing analysis (TempO-Seq). The experiments tested four chemicals (2,3-Benzofluorene, 6:1 fluorotelomer alcohol, 10:2 fluorotelomer alcohol, and perfluorohexanesulfonamide) that were selected from a list of data-poor compounds identified as high priority by the EPA (Kim et al., 2011; Li et al., 2020; Costello et al., 2022; Sen et al., 2022). Here, we used the results for the whole transcriptome that was extrapolated from the targeted RNA-sequencing platform. We analyzed the gene expression responses of these four chemicals to identify the sex-dependent molecular mechanisms by which PFAS and PAH induce a steatosis outcome in rat livers, including the key MIEs and intermediate genes. Some of the mechanisms involved disruption of a higher number of genes in male rats than in female rats and some even at doses lower than the minimum dose that induced gene disruptions in female rats. We identified the peroxisome proliferator-activated receptor (PPAR)- and hepatocyte nuclear factor 4α (HNF4 α )-mediated pathways in our study, consistent with previously reported mechanisms in mouse and human hepatocytes (Takacs and Abbott, 2007; Wolf et al., 2008; Beggs et al., 2016; Louisse et al., 2023). By analyzing an independent but similar dataset from male Sprague Dawley rats exposed to PFOA (Gwinn et al., 2020), we found supporting evidence for the expression of the genes that we identified and determined to be sex- and toxicant class-specific. Our results show that PFAS chemicals exhibit sex-specific differences in the molecular mechanisms by which they might activate a steatosis AOP. Specifically, we observed dysregulation of certain key genes belonging to the bile secretion pathway. Overall, the results suggest that the PPAR signaling and bile secretion pathways potentially play important roles in the sex-dependent activation of a steatosis AOP for PFAS exposures. Finally, the approach employed in this study can be applied to determine the activation of steatosis AOP mechanisms in any liver gene expression dataset and the lowest dose at which an activation response occurs in the case of toxicant exposure.

## 2 Materials and methods

### 2.1 Chemical selection rationale

Auerbach et al. performed experiments with four chemicals [2,3-Benzofluorene, 6:1 fluorotelomer alcohol (FTOH), 10:2 FTOH, and perfluorohexanesulfonamide (PFHxSAm)] after selecting them from a list of data-poor compounds identified by the EPA and published their data as part of NIEHS reports (Auerbach et al., 2023a; Auerbach et al., 2023b; Auerbach et al., 2023c; Auerbach et al., 2023d). Prior to these experiments, existing literature lacked *in vivo* toxicological information on these four chemicals, and there were no quantitative risk assessment values for these chemicals according to the EPA CompTox Chemicals Dashboard (United States Environmental Protection Agency, 2022; United States Environmental Protection Agency, 2023; United States Environmental Protection Agency, 2024a). We wanted to analyze the potential of these compounds to cause hepatic steatosis particularly to provide toxicity data on these understudied chemicals.

### 2.2 Animal exposure experiments

All the experiments were performed by Auerbach *et al.* using male and female Sprague Dawley rats, and detailed descriptions of the experimental study design and protocols have been published in previous NIEHS reports (Auerbach et al., 2023a; Auerbach et al., 2023b; Auerbach et al., 2023c; Auerbach et al., 2023d). Briefly, 6- to 7-week-old male and female Sprague Dawley (Hsd:Sprague Dawley) rats were obtained from Envigo (Haslett, MI) and, after a quarantine period, randomly assigned to one of 10 dose groups for each chemical (n = 5 for each sex in each dose condition; n = 10 controls for each sex). 2,3-Benzofluorene, 6:1 FTOH, and PFHxSAm were formulated at doses of 0.15, 0.50, 1.40, 4, 12, 37, 111, 333, or 1,000 mg/kg and 10:2 FTOH at doses of 0.07, 0.20, 0.70, 2, 6, 18, 55, 160, or 475 mg/kg. Vehicle controls included corn oil (2,3-Benzofluorene and 6:1 FTOH) and acetone:corn oil (1:99) (PFHxSAm and 10:2 FTOH). The chemicals 10:2 FTOH and PFHxSam were obtained from SynQuest Laboratories Inc. (Alachua, FL) while 6:1 FTOH and 2,3-Benzofluorene were obtained from Apollo Scientific, Ltd. (Stockport, UK) and Finetech Industry Limited (London, UK), respectively. The chemicals were tested for purity using gas chromatography and/or mass spectrometry. All chemicals had a purity ≥95%, with 6:1 FTOH having the highest purity (99%), followed by 2,3-Benzofluorene (98.7%), 10:2 FTOH (97.8%), and PFHxSAm (95%). To select the dose levels for each chemical, median lethal dose (LD_50_) predictions were obtained from the OPEn structure-activity/property Relationship App (OPERA) (Mansouri et al., 2018). The rats in each dose group received one of the four study chemicals or vehicle (control group) by oral gavage for 5 consecutive days (days 0–4). On day 5, in random order, the rats were euthanized by CO_2_/O_2_ (70%/30%) anesthesia, and samples from the left liver lobe were collected within 5 min. About 250 mg of each tissue were cryopreserved in RNA*later* until processing for RNA isolation.

Total RNA was extracted from the cryopreserved liver samples using RNeasy 96 QIAcube HT kits (catalog no. 74171, Qiagen Inc., Valencia, CA). After RNA purity and quality checks, the samples were stored until processing for sequencing using the rat S1500^+^ TempO-Seq platform (Yeakley et al., 2017; Mav et al., 2018). For sequencing, 1 μL of each RNA sample was hybridized with the S1500^+^ beta detector oligo pool mix followed by nuclease digestion and polymerase chain reaction (PCR) amplification. The PCR amplification products were cleaned using a PCR clean-up kit (Machery-Nagel, Mountain View, CA) before sequencing on a HiSeq 2500 Sequencing System (Illumina, San Diego, CA). Sequencing data were processed using Illumina’s BCL2FASTQ software with all parameters set to default.

The TempO-Seq sequences were then aligned to the probe sequences from the target platform using Bowtie version 1.2.2 (Langmead et al., 2009). Samples were filtered out if they had values below the following thresholds: sequencing depth <300K, total alignment rate <40%, unique alignment rate <30%, number of aligned reads <300K, or percentage of probes with at least five reads <50%. All liver samples met the inclusion criteria. Outliers were removed following identification by principal component, hierarchical cluster, and inter-replicate correlation analyses. Unattenuated equivalent counts were calculated using the attenuation factors provided in the platform documentation and were subsequently normalized at the probe level by applying reads per million normalization. Following normalization, a pseudo-count of one was added to each normalized expression value, and these values were log2 transformed. In this study, we used the normalized log-transformed values from the S1500^+^ dataset and extrapolated them to the whole transcriptome (∼17K genes) using a principal component regression (Jolliffe, 1982) approach implemented in GeniE version 3.0.4 (Sciome, 2022). The extrapolation training data included publicly available Affymetrix GeneChip Rat Genome 230 2.0 Microarray samples (∼40K) from Gene Expression Omnibus (GEO) and the Open TG-GATES Database (Igarashi et al., 2015).

Using the extrapolated log-transformed whole transcriptome (∼17K genes) dataset, we identified differentially expressed genes per dose by performing a one-way analysis of variance (ANOVA) as implemented in the anovan function in the Statistics and Machine Learning Toolbox (v12.3) on MATLAB R2022a and then estimating the false discovery rate to correct for multiple comparisons. We used the resulting gene fold change values for all the subsequent analyses.

### 2.3 Selection of MIEs and target genes

We used the webtool ToxProfiler to select the potential protein targets that act as MIEs for steatosis (AbdulHameed et al., 2021). ToxProfiler uses the structure of a chemical to predict the probability of it binding to an established toxicity target. Therefore, we provided the structures of the PFAS and PAH chemicals administered in the rat experiments as input to ToxProfiler. To test if functional group attachments affect target binding, we added additional PFAS compounds to the input list. Supplementary Table S1 in Supplementary Material S1 contains the list of compounds and their SMILES that we input to ToxProfiler. We identified additional nuclear receptor MIEs from published steatosis AOPs reviewed by Mellor et al. (Mellor et al., 2016). Furthermore, we also included additional steatosis-specific MIEs by mining the EPA-AOP database (EPA-AOP DB) (Mortensen et al., 2021) by searching for AOPs with “steatosis” or “fatty liver” in the name field. We then extracted MIEs for these AOPs from the AOP-Wiki (Society for Advancement of AOPs, 2012) using the corresponding AOP ID. We provide a complete list of MIEs identified from EPA-AOP DB and AOP-Wiki in Supplementary Datasheet S1.

To collect the genes that are targets of the potential MIEs, we used the TRRUST database (Han et al., 2018), which is a curated repository of human and mouse transcription factor and target interactions, and downloaded the table of human transcription factor and regulatory interactions, which contains genes that act as transcription factors mapped to their target genes and the type of interaction (activation, repression, or unknown). From the downloaded table, we extracted targets of the 28 MIEs identified in the previous step and converted their gene symbols to rat gene symbols using online DAVID gene ID conversion tool (Huang et al., 2008). We counted the number of activated genes for each MIE at each toxicant dose based on whether a gene was up- or downregulated with a log2 fold change (FC) value of ≥0.6 or ≤ −0.6, respectively. Furthermore, we considered gene responses to be dose-based if there was an observed increase in the FC value in the same direction (up- or downregulation) as the increase in the toxicant dose. This dose association is reported only based on the observed trend in the direction of the FC.

### 2.4 Visual analysis of MIE-target gene network

We first generated an Excel table containing columns of source nodes (MIEs) and target nodes (target genes) and imported it into the Cytoscape desktop app (Shannon et al., 2003). We provide this table as part of Supplementary Datasheet S1. We applied the default and automatic preferred layout to visualize the MIE-gene network. We used dark blue squares to represent the MIE transcription factors and circle nodes to represent the target genes. To further visualize network-level dysregulation, we imported the gene expression FC values for the target genes at the highest dose level of a toxicant in our study. We then used the FC values to color the target nodes as either a green node to indicate negative FC or downregulation of the gene or a red node to indicate positive FC or upregulation. We have also visualized this information as a heatmap (Supplementary Datasheet S2) to trace gene patterns that showed sex- or toxicant class-dependent expression.

### 2.5 Software

We used the Statistics and Machine Learning Toolbox (v12.3) to perform the ANOVA, using the anovan function, on MATLAB R2022a (MathWorks Inc, 2022), the Cytoscape desktop app (Shannon et al., 2003) for network visualizations, and the pandas (The Pandas Development Team, 2023), numpy (Harris et al., 2020), seaborn (Waskom, 2021), and matplotlib (Hunter, 2007) packages for data analysis and heatmap visualizations on Python 3.10.

## 3 Results

### 3.1 Identification of steatosis MIEs and their target genes in the exposure dataset

**FIGURE 1:**
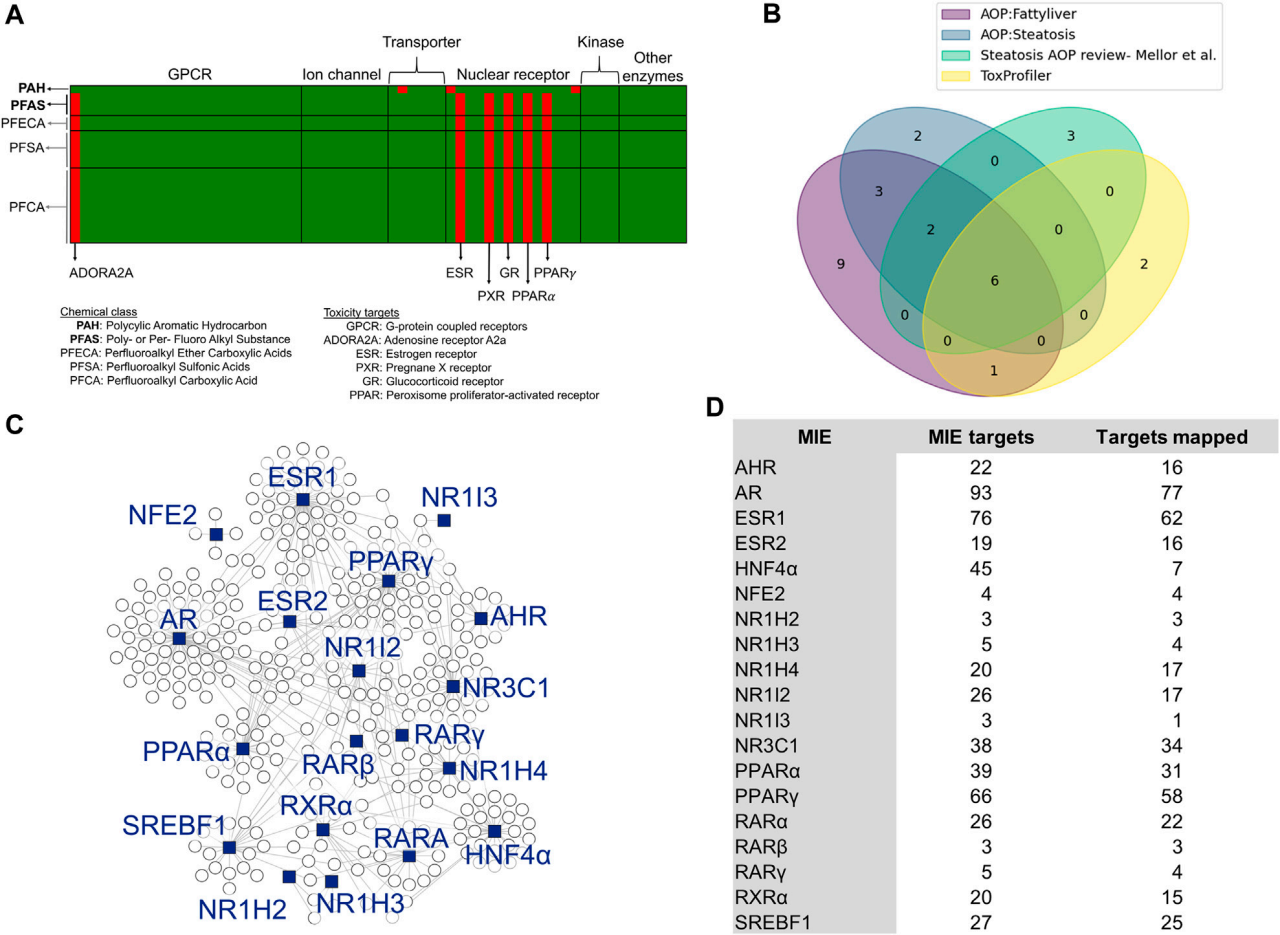
Selection of molecular initiating events (MIEs) and their target genes. **(A)** Binding targets of PFAS and PAH chemicals as predicted using ToxProfiler. Each row represents a chemical, and each column represents a toxicity target. Red cells denote a high probability of binding between a chemical and a toxicity target, such as ESR, PXR, and GR for PFAS compounds. **(B)** Overlap of MIEs collected from different sources. **(C)** Network visualization of steatosis-associated MIEs analyzed in this study and their target genes. The blue squares represent MIEs acting as transcription factors to the genes represented by the circle nodes. **(D)** List of MIEs analyzed in this study, the number of genes they modulate, and the number of those genes that map to the rat PFAS and PAH exposure dataset.

To identify MIEs associated with PFAS, we first performed a chemical structure-based prediction of protein targets for toxicity using the webtool ToxProfiler. Figure 1A shows the ToxProfiler predictions in the form of a matrix, with rows representing chemicals and columns representing toxicity targets, and lists the three PFAS and one PAH that were part of the 5-day rat exposure study (indicated in bold font) as well as a few additional PFAS chemicals that we included to determine the effect of functional group attachment on the binding of PFAS to toxicity targets (see Supplementary Material S1, Supplementary Table S1). Our results showed that all PFAS compounds have a consistent binding profile (predominantly nuclear receptors) independent of the functional group attachments or the chain length and that this binding profile is different from that predicted for the PAH. We then augmented the ToxProfiler results with MIE information from the US EPA-AOP DB (Mortensen et al., 2021) and AOP-Wiki (Society for Advancement of AOPs, 2012). The EPA-AOP DB search returned 52 AOPs for “fatty liver” and 11 AOPs for “steatosis.” We then extracted MIEs for these AOPs from AOP-Wiki and obtained 25. Since the ToxProfiler results included many nuclear receptors, we checked the literature for the association between nuclear receptors and steatosis and identified a review paper by Mellor *et al.* (Mellor et al., 2016) that lists and describes nuclear receptors that can act as MIEs in steatosis. We integrated this reviewed list with our MIEs from ToxProfiler and AOP-Wiki. The final list included 28 MIEs, which are listed with their sources in Supplementary Datasheet S1. Figure 1B shows the sources of MIEs and the number of MIEs common between the sources in the form of a Venn diagram. There were 6 MIEs that were common to all sources: AHR, ESR1/ESR2, GR/NR3C1, PPARγ, PPARα, and PXR. We collected the target genes associated with the 28 transcription factors that act as MIEs from the TRRUST database and then mapped them to the rat 5-day exposure data. Out of the 28 MIEs identified, 19 mapped to transcription factors annotated in the TRRUST database. Figure 1C shows a summary of the final list of MIEs that act as transcription factors and the genes they modulate as a network diagram. Most of the target genes (indicated as white circles) clustered around their transcription factors (indicated as blue squares) except for genes modulated by more than one transcription factor. Figure 1D shows the list of MIEs, the number of target genes for each MIE, and the number of target genes mapped to the rat 5-day exposure data. The MIEs androgen receptor (AR) and estrogen receptor 1 (ESR1) mapped to a high number of genes in the rat exposure data since they have many targets. We used the genes mapped in this section for our further analysis.

We counted the target genes that were either upregulated (FC ≥ 0.6) or downregulated (FC ≤ −0.6) for each MIE to identify exposure-induced toxicity mechanisms. Table 1 shows the number of target genes dysregulated for each MIE at each toxicant dose. The results show that most of the targets modulated by PPAR were dysregulated upon exposure to toxicants. A closer look at the number of dysregulated MIE targets revealed that most of the PPAR targets were upregulated while the HNF4α targets were downregulated (Supplementary Material S1, Supplementary Table S2). Following the PPAR targets, nuclear receptor subfamily 3 group C member 1 (NR3C1), ESR1, and AR modulated the greatest number of dysregulated targets. In addition, we found that the number of dysregulated genes was generally higher in male rats compared to female rats exposed to the same dose of toxicant. Furthermore, male rats showed dysregulation responses at doses lower than the first dose at which female rats showed any dysregulation. For example, female rat livers exhibited dysregulation of only one or two targets of PPARα and PPARγ in response to 111 mg/kg 6:1 FTOH while male rat livers showed a dysregulation of 6–11 targets in response to 37 mg/kg of the same toxicant. The PAH chemical 2,3-Benzofluorene induced dysregulation of the least number of target genes compared to the other toxicants. Out of the three PFAS, PFHxSAm did not affect as many genes in female rats as in male rats, and 10:2 FTOH induced the lowest number of MIE target dysregulations in both male and female rats.

**TABLE 1:** Number of target genes disrupted (FC ≤ −0.6 or FC ≥ 0.6) for each MIE across three highest doses of PFAS chemicals (6:1 FTOH, 10:2 FTOH, and PFHxSAm) and a PAH chemical (2,3-Benzofluorene) exposures for male and female rats. MIEs were obtained from ToxProfiler and published AOPs and their target genes were obtained from TRRUST database.

	2,3-Benzofluorene						6:1 FTOH						10:2 FTOH						PFHxSAm					
	Female			Male			Female			Male			Female			Male			Female			Male		
Dose (mg/kg)	111	333	1,000	111	333	1,000	111	333	1,000	37	111	333	55	160	475	55	160	475	12	37	111	12	37	111
AHR	3	3	4	6	5	7	0	4	9	3	7	10	2	2	2	5	5	7	0	0	3	2	3	4
AR	0	2	1	2	3	2	0	1	5	2	3	6	3	5	5	2	3	9	0	0	3	1	2	5
ESR1	0	3	4	2	2	3	1	3	11	3	6	12	3	3	1	1	5	9	0	0	4	2	2	5
ESR2	0	1	1	0	0	0	0	1	1	0	1	3	1	1	1	0	1	2	0	0	2	0	1	1
HNF4α	0	1	2	2	1	3	1	2	3	4	8	7	2	4	3	0	1	3	0	2	3	2	4	7
NFE2	0	1	1	0	0	0	0	0	0	0	0	0	0	0	1	0	0	1	0	0	0	0	0	0
NR1H2	0	0	0	0	0	0	0	0	0	0	0	0	0	0	0	0	0	0	0	0	0	0	0	0
NR1H3	0	0	1	0	0	0	0	0	0	0	0	0	0	0	0	0	0	0	0	1	1	0	0	1
NR1H4	1	1	1	1	0	0	1	1	2	1	4	3	0	1	1	0	0	3	0	0	1	0	1	2
NR1I2	1	1	1	1	2	1	0	2	2	0	3	3	0	1	1	1	1	3	0	0	1	0	0	3
NR1I3	1	1	1	1	1	1	0	1	1	0	1	1	0	0	0	1	1	1	0	0	1	0	0	0
NR3C1	1	2	2	2	2	3	0	2	6	0	3	6	1	1	2	2	1	5	0	0	2	1	1	3
PPARα	2	2	4	3	2	5	3	14	17	12	19	20	0	2	1	2	8	10	0	1	5	2	5	18
PPARγ	2	4	5	2	2	3	2	8	16	7	11	18	2	3	7	1	5	10	0	0	4	2	2	9
RARα	2	2	3	2	1	2	0	4	5	0	4	4	2	2	4	1	2	2	0	1	4	0	0	4
RARβ	0	0	0	0	0	0	0	0	0	0	2	2	0	0	0	0	0	1	0	0	0	0	0	1
RARγ	0	0	0	0	0	0	0	0	1	0	1	1	0	0	1	0	1	1	0	0	0	0	0	1
RXRα	2	2	2	2	1	1	1	2	2	0	3	2	1	2	2	1	1	2	0	0	2	0	1	3
SREBF	1	0	1	3	4	3	2	6	9	4	5	7	1	0	5	2	2	3	1	0	3	4	1	6

PAH, polycyclic aromatic hydrocarbons; PFAS, per- or polyfluoro alkyl substances; FC, fold change; FTOH, fluorotelomer alcohol; MIE, molecular initiating event; PFHxSAm, perfluorohexanesulfonamide; AHR, aryl hydrocarbon receptor; AR, androgen receptor; ESR1/2, estrogen receptor 1/2; HNF4α, hepatocyte nuclear factor 4α; NFE2, nuclear factor erythroid 2; NR1H2/3/4, nuclear receptor subfamily 1, group H, member 2/3/4; NR1I2, nuclear receptor subfamily 1, group I, member 2; NR3C1, nuclear receptor subfamily 3, group C, member 1; PPARα/γ, peroxisome proliferator-activated receptor α/γ; RAR, retinoic acid receptor; RXR, retinoid X receptor; SREBF1, sterol regulatory element binding transcription factor 1.

**FIGURE 2:**
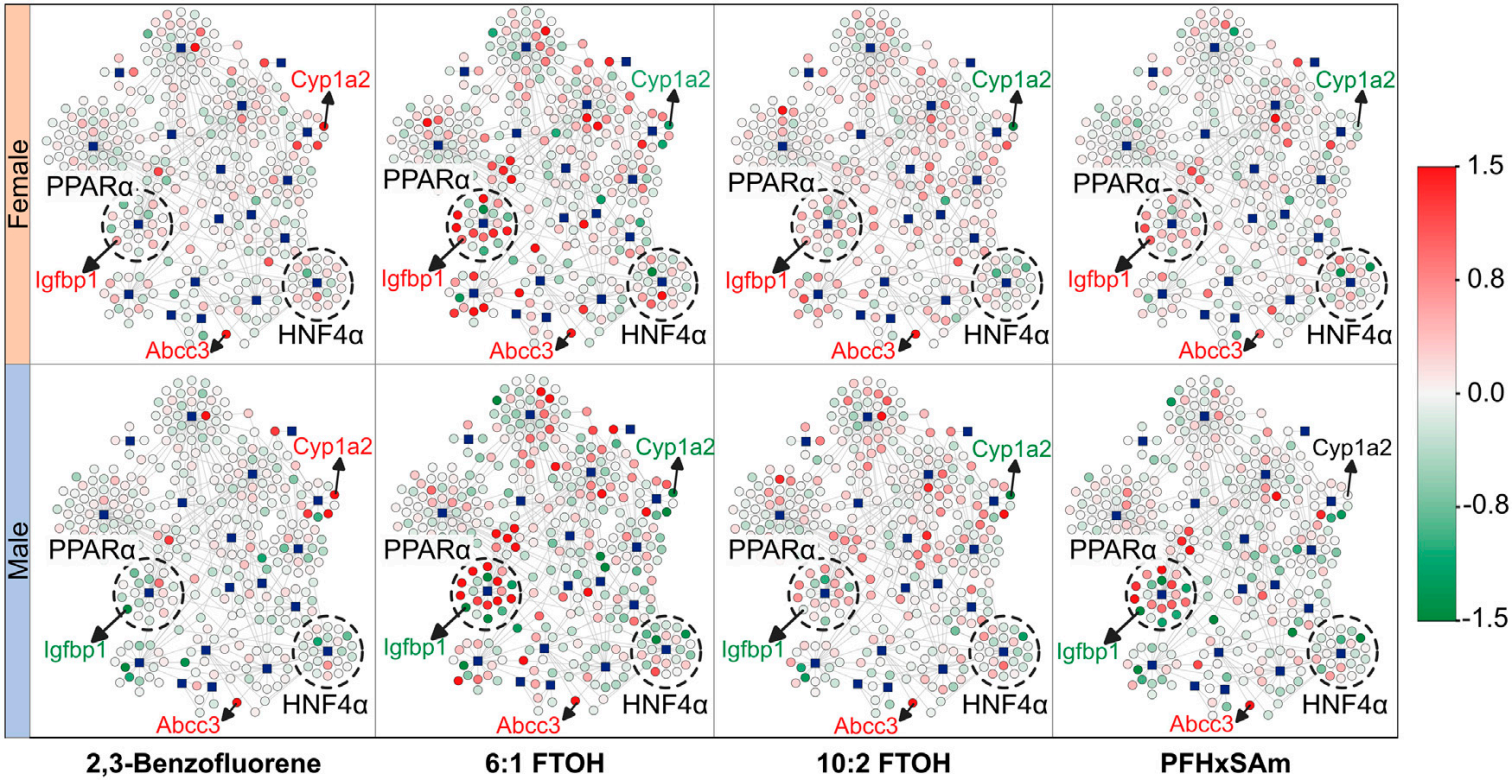
Molecular initiating event (MIE)-target gene network showing the expression response in male and female rats after exposure to the highest dose of each toxicant. The blue square nodes represent the MIEs, and the circle nodes denote the target genes. Nodes in green indicate downregulation of the gene [log fold change (FC) ≤ 0.0], and nodes in red indicate upregulation of the gene (log (FC) ≥ 0.0). Labeled genes represent examples of genes with expression independent of rat sex or toxicant class (*Abcc3*) and genes with expression dependent on sex (*Igfbp1*) or toxicant class (*Cyp1a2*). Dashed circles highlight MIEs and their targets that were found to be predominantly up- (PPARα) or downregulated (HNF4α) due to toxicant exposure. MIEs were obtained from ToxProfiler and published AOPs and their target genes were obtained from TRRUST database.

To understand the overall expression patterns of the mapped genes that indicate activation of MIEs leading to steatosis, we superimposed the FC values of the genes from the rat PFAS and PAH exposure data on the MIE-target gene network in Figure 1C. The resultant network diagrams and genes dysregulated at the highest dose of each toxicant are shown in Figure 2, with nodes in green indicating downregulation of the gene (FC ≤ −0.6) and nodes in red indicating upregulation (FC ≥ 0.6). Our results here again suggested that the male rats showed disruption of a higher number of target genes compared to the female rats. Furthermore, we could visualize that 6:1 FTOH was the most potent chemical and disrupted the highest number of genes, in particular upregulating many of the PPARα targets. The expression of these genes in the form of a heatmap (Supplementary Datasheet 2) further helped identify genes that *1*) responded identically across all the chemicals (consistent up- or downregulation), *2*) showed a PFAS- or PAH-specific response, or *3*) showed a sex-specific response. For example, we identified that the gene *Abcc3* was consistently upregulated in response to all the chemicals irrespective of sex, suggesting that some of the responses are toxicant- and sex-independent. Similarly, we identified *Cyp1a2* that was upregulated on exposure to the PAH and downregulated on exposure to any of the PFAS compounds, indicating responses that differed based on the class of chemical. We also identified genes with sex-dependent responses, such as the gene *Igfbp1* that was consistently downregulated in male rats but upregulated in female rats. We labeled these nodes in the network diagrams in Figure 2. Our results indicate that sex and the class of the chemical can affect the responses of these target genes and that the variation in these gene responses may potentially differentially activate the steatosis AOP.

### 3.2 Identification of individual genes with rate-limiting function and chemical class- or sex-dependent expression

**FIGURE 3:**
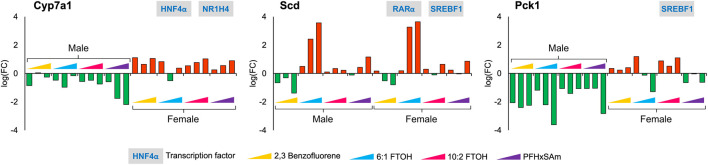
Disruption of genes with rate-limiting functions as a result of a 5-day acute exposure to three highest doses of PAH or PFAS compounds. The green and red bars denote negative and positive log fold change (FC) values indicating down- and upregulation, respectively. Each bar represents log (FC) value [FC: ratio of treatment dose group (n = 5) to the control group (n = 10)] for one of the top three doses for a given chemical as indicated in Table 1. The grey boxes with blue text contain the transcription factor(s)/MIEs (listed in Table 1) that are known to modulate the given gene.

Given the individual differences in gene expression profiles between chemicals and by sex in the heatmap (Supplementary Datasheet S2), we further explored all the target genes independently across all the doses of all four chemicals in the 5-day rat studies, for both male and female rats. Our analysis identified several genes with key functional roles that are consistently up- or downregulated for each chemical, some of which even showed a dose-based trend. These included genes that have crucial rate-limiting liver metabolic functions, such as *Pck1* in gluconeogenesis, *Cyp7a1* in bile acid synthesis, and *Scd* in the synthesis of monounsaturated fatty acids. Figure 3 shows the observed expression pattern of these genes at the three highest dose levels of each toxicant in male and female rats. The logarithmic FC values of the genes *Cyp7a1* and *Pck1* showed a sex-dependent downregulation only in male rats, whereas they were upregulated in their female counterparts. The gene *Scd,* which plays a crucial role in fatty acid metabolism, showed a PFAS-specific upregulation in both male and female rats and was downregulated in response to the PAH chemical. Furthermore, we observed a dose-based trend for *Cyp7a1* in male rats exposed to PFHxSAm and in female rats exposed to 10:2 FTOH or PFHxSAm such that the FC continuously increased (or decreased in the case of downregulation) with increasing concentrations of the chemical. Similarly, we observed a dose-based trend in the downregulation of *Pck1* in male rats (FC < −2.0), with the highest FC at the highest dose in response to all toxicants except 10:2 FTOH. However, in female rats, the expression pattern of *Pck1* was not consistent across all four of the chemicals, with inconsistent downregulation in response to 6:1 FTOH and PFHxSAm. We observed a similar behavior for the gene *Scd,* which showed increasing upregulation in response to increasing doses of 6:1 FTOH in both male and female rats, with some inconsistencies in response to 10:2 FTOH. Interestingly, two of these rate-limiting genes, *Pck1* and *Scd*, were modulated by the transcription factor SREBF1, suggesting its importance in the activation of a liver steatosis response.

**FIGURE 4:**
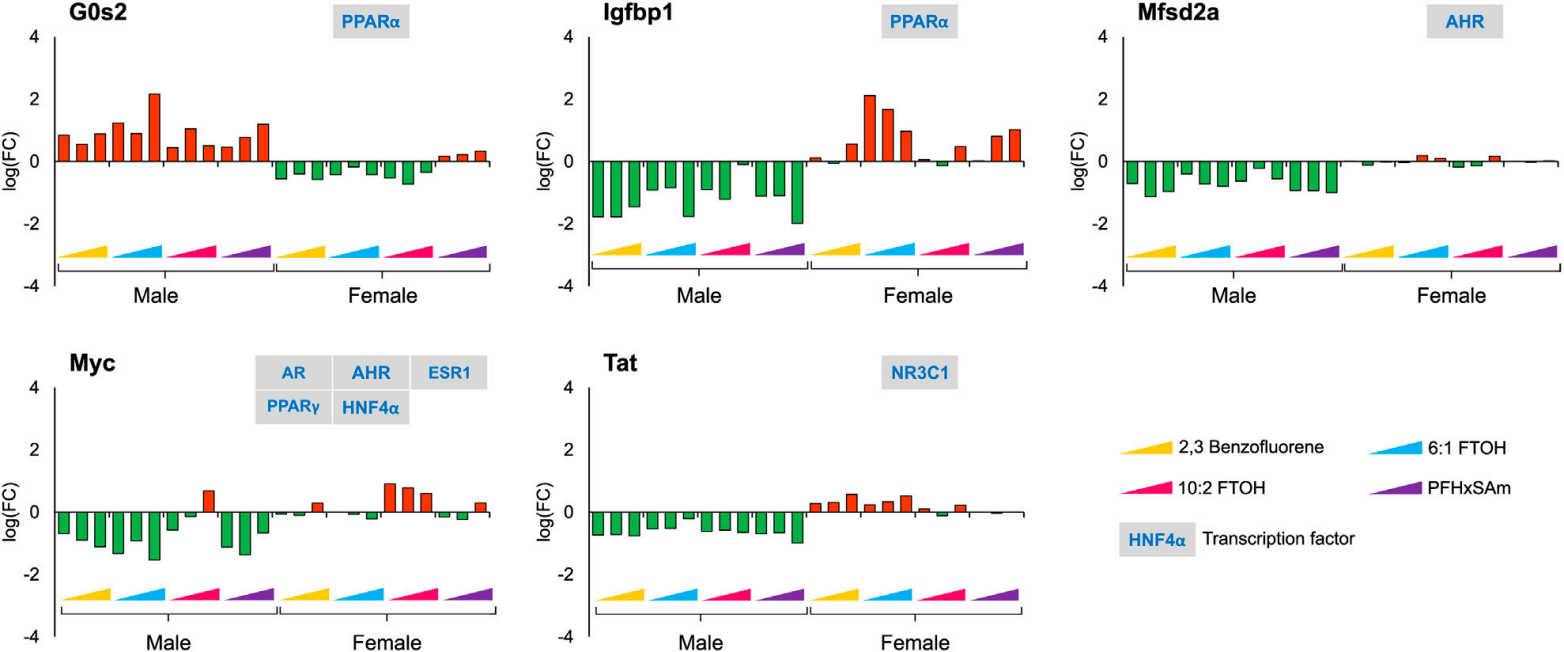
Genes with sex-specific responses to top three highest doses of both PFAS and non-PFAS toxicants. The green and red bars denote negative and positive log fold change (FC) values indicating down- and upregulation, respectively. Each bar represents log (FC) value [FC: ratio of treatment dose group (n = 5) to the control group (n = 10)] for one of the top three doses for a given chemical as indicated in Table 1. The grey boxes with blue text contain the transcription factor(s)/MIEs (listed in Table 1) that are known to modulate the given gene.

With respect to sex-dependent gene responses, our analysis of the target gene expression profiles identified several genes that showed a sex-specific response upon toxicant exposure, as shown in Figure 4. Most of the genes, except *G0s2*, were downregulated in male rats but upregulated in female rats. In male rats, the FC values of *G0s2*, *Igfbp1,* and *Myc* were higher in response to 6:1 FTOH compared to other toxicants while the FC of *Tat* was approximately the same across all toxicants.

**FIGURE 5:**
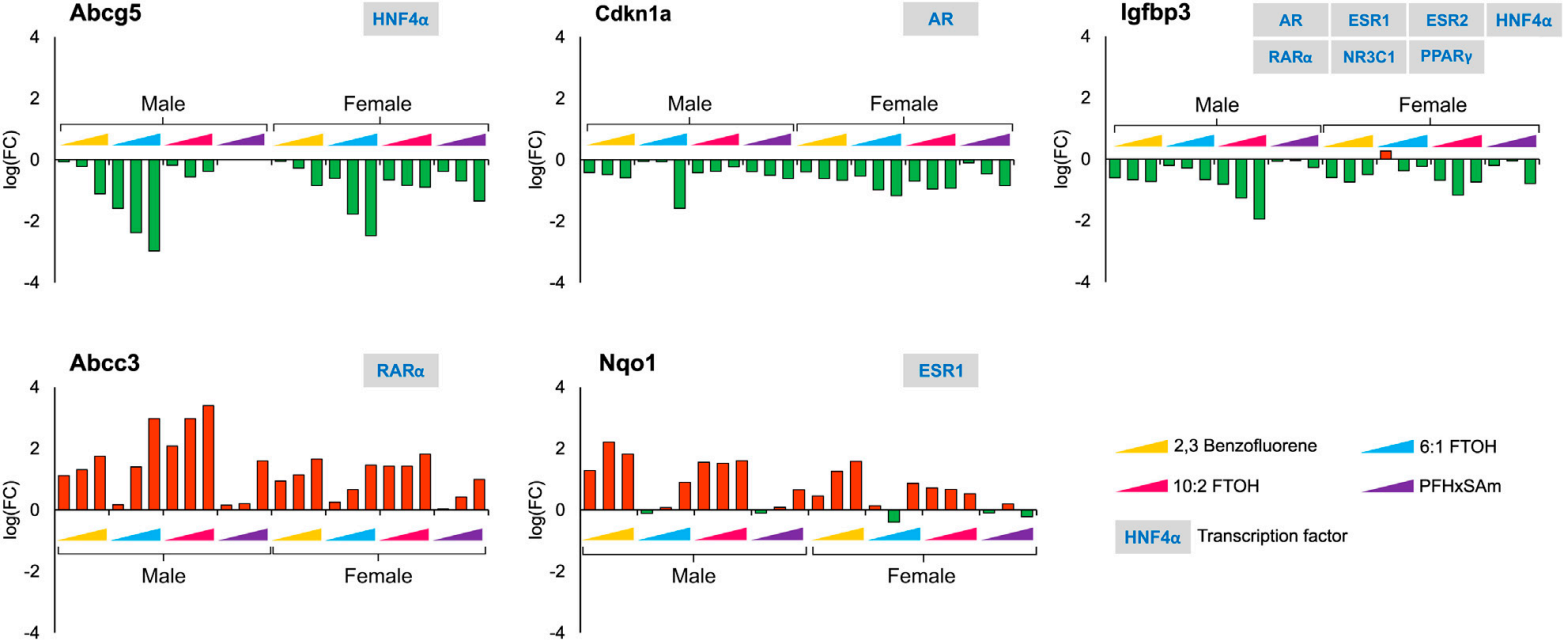
Genes that showed a similar response across all four toxicants and between sexes after a 5-day exposure to the three highest doses of PAH or PFAS compounds. The green and red bars denote negative and positive log fold change (FC) values indicating down- and upregulation, respectively. Each bar represents log (FC) value [FC: ratio of treatment dose group (n = 5) to the control group (n = 10)] for one of the top three doses for a given chemical as indicated in Table 1. The grey boxes with blue text contain the transcription factor(s)/MIEs (listed in Table 1) that are known to modulate the given gene.

In contrast to the genes that showed sex-dependent behavior, we identified several genes that had a similar response across all the chemicals in both male and female rats. For example, Figure 5 shows the genes that had a consistent downregulation (*Abcg5, Cdkn1a,* and *Igfbp3*) or upregulation (*Abcc3* and *Nqo1*) across all toxicants and in both sexes. The transporter gene *Abcg5* that is required to eliminate cholesterol from hepatocytes is downregulated upon toxicant exposure, indicating additional potential for cholesterol accumulation. The response of *Nqo1,* a gene that is elevated in response to stress and injury in the liver (Aleksunes et al., 2006; Ross and Siegel, 2021), was different for 6:1 FTOH and PFHxSAm. Most of these genes showed dose-based responses, with the greatest response elicited by the chemical 6:1 FTOH. Furthermore, we observed a greater magnitude of change in male compared to female rats at the highest dose of exposure. We also observed that most of these genes, except *Igfbp3,* were modulated by a single transcription factor.

**FIGURE 6:**
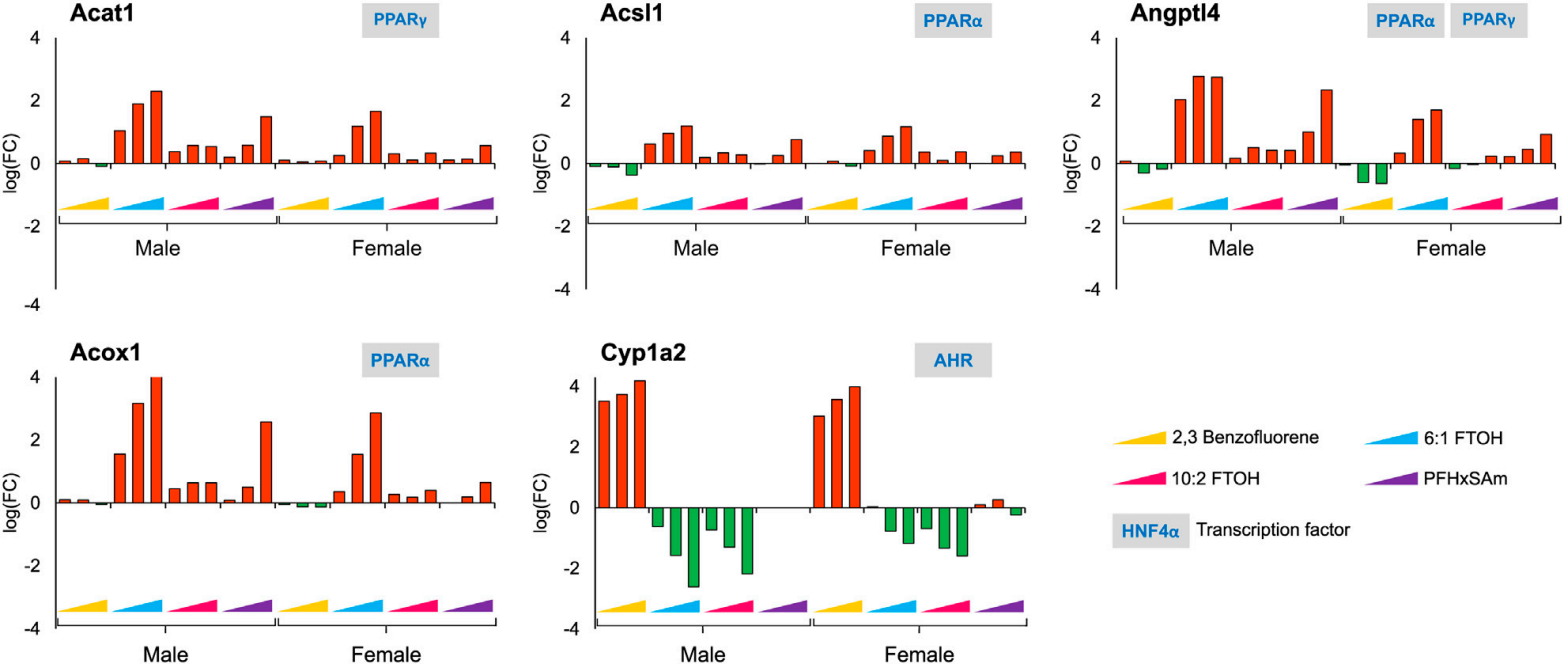
Genes that showed a similar and PFAS-specific response in both male and female rats to a 5-day acute exposure to the three highest doses of PAH or PFAS compounds. The green and red bars denote negative and positive log fold change (FC) values indicating down- and upregulation, respectively. Each bar represents log (FC) value [FC: ratio of treatment dose group (n = 5) to the control group (n = 10)] for one of the top three doses for a given chemical as indicated in Table 1. The grey boxes with blue text contain the transcription factor(s)/MIEs (listed in Table 1) that are known to modulate the given gene.

Since our study contains a combination of PFAS and PAH chemicals, we explored the expression dataset to identify target gene responses that differentiate PFAS from PAH chemicals. For example, we identified *Cyp1a2*, a key target of aryl hydrocarbon receptor (AHR), to be upregulated in response to 2,3-Benzofluorene but downregulated to the PFAS compounds. Figure 6 shows other genes, in addition to *Cyp1a2,* that respond differently to PFAS and PAH exposures, including *Acat1, Acsl1, Angptl4,* and *Acox1*. Most of these genes were upregulated in response to PFAS exposure, with the greatest dose-based response elicited by 6:1 FTOH. In addition, the observed FC values at the highest dose of each PFAS were higher in the male *versus* female rats. Interestingly, we observed that all these PFAS-specific genes are targets of either PPARα or PPARγ, indicating a major role of this transcription factor in modulating a PFAS-specific response.

### 3.3 Analysis of expression of other steatosis-associated genes

**FIGURE 7:**
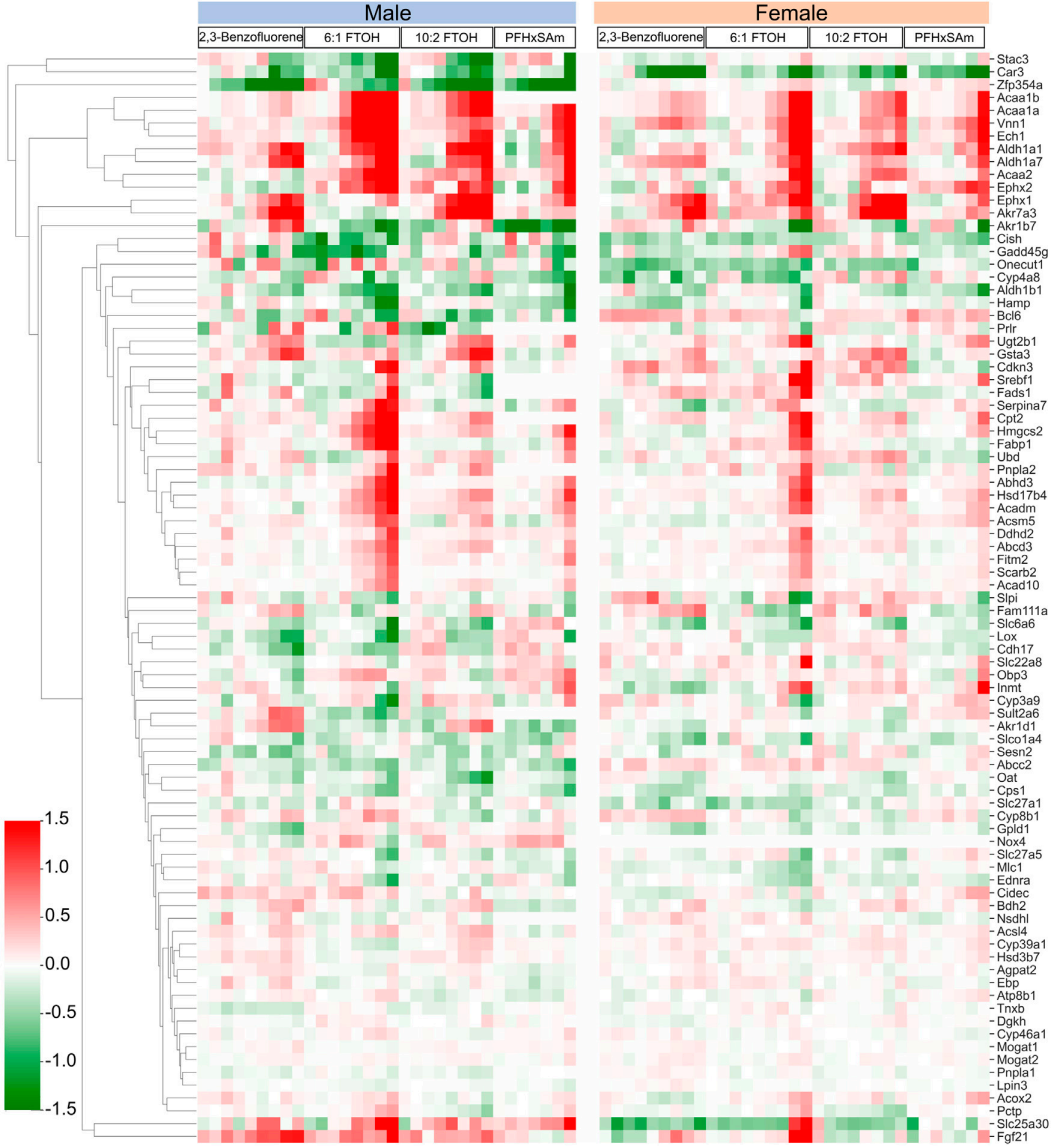
Dose response behavior of genes known to be involved in activation of steatosis AOP outcome. Logarithmic fold change expression of altered genes when male and female rats are exposed to different PFAS and PAH chemicals across various dose levels (*x*-axis represents increasing concentration of each chemical from left to right). Red cells indicate positive fold change values (upregulation) while green cells indicate negative fold change values (downregulation).

In addition to the target genes that we obtained based on the steatosis AOP MIEs as discussed above, a few studies have reported genes commonly disrupted by steatosis-inducing chemicals (AbdulHameed et al., 2019) or in response to PFAS exposure (Ankley et al., 2010; Bagley et al., 2017). Therefore, to further analyze steatosis- and PFAS-associated gene responses in the current data, we collected the genes from such studies and analyzed their expression in the current exposure dataset. The heatmap in Figure 7 shows the dose-wise expression of each of the genes, clustered by their expression. We identified a couple of genes, *Zfp354a* and *Stac3,* that were downregulated in male rats. At the highest dose, 6:1 FTOH induced maximum alterations in the FC values of most of the genes in both sexes. Several genes were upregulated across all toxicants (*Acaa1b, Acaa1a, Vnn1, Ech1, Aldh1a1, Aldh1a7, Acaa2, Ephx2, Ephx1, and Akr7a3*). One gene, *Akr1b7,* showed a PFAS-specific downregulation. A cluster of genes, which included *Cish*, *Gadd45g*, *Onecut1*, *Cyp4a8*, *Hamp*, and *Aldh1b1*, showed inconsistent responses between sexes, chemicals, and even doses of the same chemical. *Bcl6* was downregulated in male rats at the highest dose of each toxicant, whereas it was upregulated in female rats on exposure to most toxicants, except 6:1 FTOH. The genes *Gsta3*, *Cdkn3*, *Serbf1*, *Fads1*, *Serpina7*, *Cpt2*, *Hmgcs2*, *Fabp1*, *Pnpla2*, *Abhd3*, *Hsd17b4*, *Acadm*, *Acsm5*, *Ddhd2*, *Abcd3*, *Fitm2*, *Scarb2*, and *Acad10* were predominantly upregulated in response to the highest doses of PFAS, with higher FC values in male compared to female rats. However, in female rats, the FC values of these genes in response to 6:1 FTOH were comparable to those in male rats. Some transporter genes, including *Slc27a1*, *Slc27a5*, and *Oat*, were downregulated in male and female rats. The transporter gene *Abcc2* showed sex-dependent FC, but the values were low (<0.5 and > −0.5). The transporter gene *Slc25a30* was predominantly upregulated in male rats exposed to high doses of all toxicants but was mainly downregulated in female rats, except in response to 6:1 FTOH.

In another study, Natsoulis *et al.* characterized gene signatures associated with pharmacological and toxicological end points in the liver using a supervised classification approach (Natsoulis et al., 2008). We analyzed the expression of the genes that were part of the liver lipid accumulation signature, as classified in the previously mentioned study. The results of Natsoulis *et al.* associated a weight with each gene, with positive weights indicating upregulation with pathology and negative weights indicating downregulation with pathology. Using the weights, we identified the genes that were significantly dysregulated (q < 0.1) and followed the expression defined in the characterized signature or showed opposite behavior. Supplementary Datasheet S3 contains the gene sets analyzed along with their weights and expression in our dataset. We observed that while some of the genes followed the expected expression pattern (e.g., *Snx10*, *Tf*, *Hdc*, *Hamp*, and *Fam13a*) at the highest dose of each toxicant, a larger number of genes showed expression that was opposite to that in the gene signature (e.g., *Cryl1*, *Ehhadh*, *Orm1*, *Serpina6*, *Nrep*, and *Abcg5*). We obtained gene function information from the UniProt database (UniProt, 2023a) to understand the roles of the genes. The gene *Orm1* showed an increasing FC in response to increasing doses of all the toxicants, indicating an acute inflammatory response in the liver (UniProt, 2014). Similarly, *Adh5* was upregulated and mostly showed a dose-associated trend in response to all the toxicants, potentially increasing the oxidation of long-chain omega-hydroxy fatty acids and clearance of formaldehyde from the cells (UniProt, 2022). The gene *Ehhadh*, which catalyzes two reactions of long-chain fatty acid peroxisomal beta-oxidation (UniProt, 2023b), was upregulated in a dose-based, PFAS-specific manner, indicating higher fatty acid beta-oxidation in response to PFAS chemicals. These results indicate that the gene signature for PFAS- and PAH-induced lipid accumulation is potentially different from the classes of toxicants in the Natsoulis et al. study. These results also suggest the multi-factorial nature of PFAS- and PAH-induced lipid accumulation in the liver.

## 4 Discussion

**FIGURE 8:**
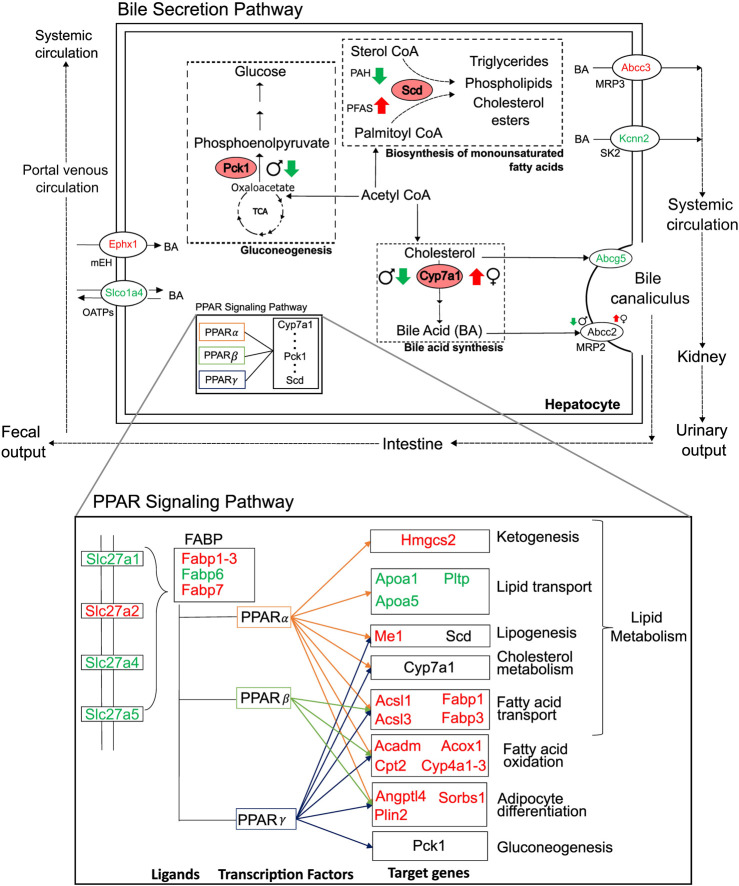
Summary of major perturbations in the lipid and bile-acid metabolism. Genes with key metabolic functions in the bile secretion and lipid metabolism pathways of the liver, as determined using the *Kyoto Encyclopedia of Genes and Genomes*. Up- and downregulated genes are indicated in red and green, respectively. Sex- and toxicant-specific responses are summarized using symbols adjacent to the gene name. Pink ovals represent genes with rate-limiting functions (*Pck1, Scd,* and *Cyp7a1*).

We analyzed liver transcriptomic data from male and female rats exposed to various doses of environmental toxicants, i.e., PFAS and PAH, to identify genes that are dysregulated and could potentially contribute to a steatosis outcome in the liver. We identified genes by grouping them into sets based on their responses: sex- and toxicant-independent, sex-dependent, or toxicant-dependent. Functionally, most of the dysregulated genes play roles in lipid metabolism and bile secretion processes (Figure 8). We observed that the key rate-limiting genes of the bile acid (*Cyp7a1*), glucose (*Pck1*), and monounsaturated fatty acid synthesis pathways (*Scd*) are disrupted in response to the toxicants and that *Pck1* and *Cyp7a1* are downregulated specifically in male rats upon exposure to the toxicants. The behavior of these genes has been previously reported to be associated with fatty liver disease in various organisms, including mice and humans (Liu et al., 2016; Chiang and Ferrell, 2020; Ye et al., 2023). From the metabolic pathway diagrams (Figure 8), we observed that the downregulation of *Pck1* reduces the transformation of oxaloacetate to phosphoenolpyruvate, which reduces cycling of acetyl CoA into glucose and makes more acetyl CoA available for cholesterol and fatty acid production. The downregulation of *Cyp7a1* reduces the production of bile acids from cholesterol, which can cause cholesterol to accumulate. The specific expression of these genes could contribute to the PFAS-induced and sex-dependent activation of steatosis observed in rodent studies (Kim et al., 2011; Roth et al., 2021; Sen et al., 2022). The up- or downregulation of additional genes that showed sex-specific responses (*G0s2, Igfbp1, Mfsd2a, Myc,* and *Tat*), as observed in the male rats, has been reported to increase lipid accumulation and lead to a non-alcoholic steatohepatitis-like phenotype (Fu et al., 2010; Wang et al., 2013; de la Garza et al., 2017; Jiang et al., 2021; Chin et al., 2023). The expression of several genes that showed a consistent response in male rats varied by dose and type of PFAS in female rats, which could further explain the contradicting results in sex-based PFAS studies (Kim et al., 2011; Roth et al., 2021; Sen et al., 2022).

While we observed that some genes respond differently between the sexes and some show a PFAS *versus* non-PFAS response, we also identified a set of genes that showed a similar response in both sexes and in response to both PFAS and non-PFAS exposures (Figures 5, 8). The upregulation of genes with fatty acid synthesis, transport, and oxidation functions and downregulation of lipid transport genes provide evidence of potential for lipid accumulation, particularly via the PPAR signaling pathway (Kanehisa and Goto, 2000; Heintz et al., 2022). We also analyzed the expression of the genes described above in an independent expression dataset of male Sprague Dawley rats exposed to various doses of PFOA (Gwinn et al., 2020) and observed that the male- and PFAS-specific responses were identical in this dataset (Supplementary Material S1; Supplementary Figure S1). Interestingly, the genes showed the adverse responses even at low doses of PFOA, which is a legacy PFAS that has been discontinued due to its high toxicity. These results suggest that the identified mechanism leading to steatosis outcome is similar among PFAS chemicals and that the length and type of PFAS likely influences the extent of disruption within the mechanism. A similar comparison would need to be performed between our results for 2,3-Benzofluorene and other PAH chemicals to make PAH-specific conclusions.

We noticed that the genes that were commonly up- or downregulated by PFAS and non-PFAS toxicants showed a lower FC in response to the non-PFAS compound. Furthermore, there were genes that showed a different response between PFAS and non-PFAS chemicals (see Figures 3, 6). Exposure to PAH caused a downregulation of *Scd*, which plays a rate-limiting function in the production of triglycerides and other fatty acids from acetyl CoA products. In contrast, exposure to PFAS caused an upregulation of *Scd*, suggesting that the adverse outcome of PFAS is dependent on increased production of fatty acids from acetyl CoA products, which could be a key step that makes the PFAS mechanism of injury different from that employed by PAH. The downregulation of *Scd* in response to PAH and upregulation in response to PFAS were similar between the sexes, with male rats showing higher FC than female rats. We also observed upregulation of *Angptl4* in rats exposed to PFAS but not in rats exposed to PAH, which indicates increased cholesterol synthesis in PFAS-exposed rats (Lichtenstein et al., 2007). These results indicate that PFAS and PAH likely activate different molecular mechanisms in steatosis adverse outcome pathways and, while the mechanism is similar among the PFAS, the magnitude of the alteration likely depends on the PFAS chain length and functional group. This finding is supported by other studies that report PFAS chain length and functional moiety affecting PFAS activity *in vivo* (Wan et al., 2012; Fenton et al., 2021).

Lipid accumulation in the liver and an increased incidence of fatty liver disease, such as hepatic steatosis, are the most commonly reported adverse outcomes of PFAS exposure (Wan et al., 2012; Beggs et al., 2016; Bagley et al., 2017; Das et al., 2017; Roth et al., 2021; Costello et al., 2022; Zhang et al., 2023). The responses of the specific genes described above have been reported to independently play roles in altering lipid composition within the liver and consequently contributing to steatosis and other liver injury phenotypes (Yu et al., 2002; Wu et al., 2004; Aleksunes et al., 2006; Li et al., 2009; Chai et al., 2012; Zhang et al., 2017; He et al., 2020; Pan et al., 2021; Chin et al., 2023). In addition to looking at genes with similar responses by toxicant or sex, we also analyzed the expression of a gene set associated with a toxicant-induced steatosis outcome (AbdulHameed et al., 2019). The results (shown in Figure 7) revealed that most of the genes in this set are disrupted more in response to PFAS than to PAH, which further suggests that PFAS chemicals likely have a greater potential to cause steatosis than PAH.

NAFLD occurs because of an imbalance between lipid uptake, biosynthesis, and elimination processes (via oxidation and export) in the liver (Tessari et al., 2009; Berlanga et al., 2014). Our analysis revealed many genes within each of the processes of lipid and fatty acid transport, lipogenesis, and fatty acid oxidation that could disrupt lipid homeostasis in the liver. Our results suggest that the steatosis outcome from the PFAS exposures could be multifactorial, with several genes in the lipid uptake, transport, and biosynthesis steps dominating the overall outcome compared to the protective upregulation of fatty acid oxidation-related genes driven by the PPAR signaling pathway. However, our analysis of transcriptomics data alone is insufficient to quantitatively identify the key driver of lipid imbalance caused by toxicant exposure. While the clinical chemistry data from the NIEHS studies (Auerbach et al., 2023a; Auerbach et al., 2023b; Auerbach et al., 2023c; Auerbach et al., 2023d), corresponding to the transcriptomics data analyzed here, showed an increase in ALT and AST levels (Supplementary Material S1, Supplementary Table S3), which provides indirect evidence of liver injury, a histological analysis would be required to confirm the steatosis outcome. ALT and AST levels were both increased in male and female rats exposed to the fluorotelomer alcohols (6:1 FTOH and 10:2 FTOH), and only ALT increased in response to PFHxSAm, suggesting varying potencies between the PFAS types. Interestingly, cholesterol levels showed a PAH-specific increase, while an increase in triglyceride levels was observed mostly in female rats.

In this study, we used published steatosis AOPs and predictions from ToxProfiler to identify molecular events that initiate a steatosis outcome in rats exposed to PFAS or PAH (Figure 1). Our results revealed that most of the disrupted genes are targets of PPAR, followed by glucocorticoid receptor (NR3C1), AR, and ESR1 (Table 1). Previous studies have reported PPAR-dependent and -independent mechanisms of PFAS toxicity in mouse, rat, and human hepatocytes (Takacs and Abbott, 2007; Wolf et al., 2008; Elcombe et al., 2012; Rosen et al., 2017; Heintz et al., 2022; Louisse et al., 2023). Some studies have also reported HNF4α-dependent mechanisms (Beggs et al., 2016) and estrogen receptor-mediated mechanisms (Behr et al., 2018; Qiu et al., 2020). Our results indicate that PFAS may be using estrogen signaling in addition to PPAR signaling to cause adverse outcomes, but the exact mechanism of toxicity may depend on the length and type of PFAS. Carboxylate-based PFAS have been reported to be more potent at activating PPAR signaling mechanisms than sulfonic acid-based PFAS (Wolf et al., 2008). We observed a similar result in the rat expression data we analyzed, suggesting that functional group attachment and length of PFAS need to be considered in studies analyzing the toxic effects of PFAS. Furthermore, the MIEs and genes studied here could be associated with outcomes other than steatosis. For example, our search of alternative AOPs on the EPA AOP-DB for the same set of genes revealed associations with reproductive dysfunction and early life stage mortality, with steatosis as one of the major outcomes, as shown in Supplementary Material S1, Supplementary Figure S2. Interestingly, some of the alternative outcomes (Leydig cell tumors, impaired fertility via malformation of the male reproductive tract, and decreased fertility via adult Leydig cell dysfunction) were also specific to male rats.

In conclusion, the widespread occurrence of toxicants that can bioaccumulate is a cause of concern due to their long half-lives and ability to rapidly accumulate in organisms (Fenton et al., 2021; Mallah et al., 2022). Here, we studied the potential of a limited set of PFAS and PAH chemicals to independently cause steatosis adverse outcomes in rats at various doses and during acute exposure. Although the FC values are not alarmingly high for the highest dose of any of the toxicants, it is possible that the toxicants induce more drastic effects on human livers due to species-specific metabolic differences or have a higher affinity for the human isoforms of the MIEs (Wolf et al., 2008; Qiu et al., 2020). The genes that we report to show dose-based trends were identified from observed FC responses, and further analysis that includes dose-response modeling would be needed to evaluate the significance of dose-dependency. The amount of data analyzed here was large, with over 350 samples across ∼17K genes, from which we extracted key sex-based differences that could potentially activate a steatosis outcome. While our approach does not describe the potential of PFAS to cause steatosis specifically in humans, we highlight genes and mechanisms that can be translated to understand adverse outcomes in humans and to design therapeutic approaches that can circumvent the toxic effects of PFAS. Finally, our approach can be applied to computationally design and analyze steatosis AOPs for various toxicants using any liver gene expression data.

## Data Availability

Publicly available datasets were analyzed in this study. This data can be found here: GEO database accession no. GSE183705.

## References

[B1] AbdulHameed M. D. M. Liu R. Schyman P. Sachs D. Xu Z. Desai V. (2021). ToxProfiler: toxicity-target profiler based on chemical similarity. Computat. Toxicol. 18, 100162. 10.1016/j.comtox.2021.100162

[B2] AbdulHameed M. D. M. Pannala V. R. Wallqvist A. (2019). Mining public toxicogenomic data reveals insights and challenges in delineating liver steatosis adverse outcome pathways. Front. Genet. 10, 1007. 10.3389/fgene.2019.01007 31681434 PMC6813744

[B3] Aleksunes L. M. Goedken M. Manautou J. E. (2006). Up-regulation of NAD(P)H quinone oxidoreductase 1 during human liver injury. World J. Gastroenterol. 12 (12), 1937–1940. 10.3748/wjg.v12.i12.1937 16610002 PMC4087521

[B4] Ankley G. T. Bennett R. S. Erickson R. J. Hoff D. J. Hornung M. W. Johnson R. D. (2010). Adverse outcome pathways: a conceptual framework to support ecotoxicology research and risk assessment. Environ. Toxicol. Chem. 29 (3), 730–741. 10.1002/etc.34 20821501

[B5] Attanasio R. (2019). Sex differences in the association between perfluoroalkyl acids and liver function in US adolescents: analyses of NHANES 2013-2016. Environ. Pollut. 254 (Pt B), 113061. 10.1016/j.envpol.2019.113061 31454574

[B6] Auerbach S. S. (2023a) NIEHS report on the *in vivo* repeat dose biological potency study of 2,3-Benzofluorene (CASRN 243-17-4) in Sprague Dawley (Hsd:Sprague Dawley® SD®) rats (gavage studies): NIEHS report 09. Research Triangle Park, NC: NIEHS. 37018437

[B7] Auerbach S. S. (2023b) NIEHS report on the *in vivo* repeat dose biological potency study of 6:1 Fluorotelomer Alcohol (CASRN 375-82-6) in Sprague Dawley (Hsd:Sprague Dawley® SD®) rats (gavage studies): NIEHS report 07. Research Triangle Park, NC: NIEHS. 37018435

[B8] Auerbach S. S. (2023c) NIEHS report on the *in vivo* repeat dose biological potency study of 1,1,2,2-Tetrahydroperfluoro-1-dodecanol (CASRN 865-86-1) in Sprague Dawley (Hsd:Sprague Dawley® SD®) rats (gavage studies): NIEHS report 08. Research Triangle Park, NC: NIEHS. 37018436

[B9] Auerbach S. S. (2023d) NIEHS report on the in vivo repeat dose biological potency study of perfluorohexanesulfonamide (CASRN 41997-13-1) in Sprague Dawley (Hsd:Sprague Dawley® SD®) rats (gavage studies): NIEHS report 10. Research Triangle Park, NC: NIEHS. 37018438

[B10] Bagley B. D. Chang S. C. Ehresman D. J. Eveland A. Zitzow J. D. Parker G. A. (2017). Perfluorooctane sulfonate-induced hepatic steatosis in male Sprague Dawley rats is not attenuated by dietary choline supplementation. Toxicol. Sci. 160 (2), 284–298. 10.1093/toxsci/kfx185 28973659

[B11] Beggs K. M. McGreal S. R. McCarthy A. Gunewardena S. Lampe J. N. Lau C. (2016). The role of hepatocyte nuclear factor 4-alpha in perfluorooctanoic acid- and perfluorooctanesulfonic acid-induced hepatocellular dysfunction. Toxicol. Appl. Pharmacol. 304, 18–29. 10.1016/j.taap.2016.05.001 27153767 PMC5367386

[B12] Behr A. C. Lichtenstein D. Braeuning A. Lampen A. Buhrke T. (2018). Perfluoroalkylated substances (PFAS) affect neither estrogen and androgen receptor activity nor steroidogenesis in human cells *in vitro* . Toxicol. Lett. 291, 51–60. 10.1016/j.toxlet.2018.03.029 29601859

[B13] Berlanga A. Guiu-Jurado E. Porras J. A. Auguet T. (2014). Molecular pathways in non-alcoholic fatty liver disease. Clin. Exp. Gastroenterol. 7, 221–239. 10.2147/CEG.S62831 25045276 PMC4094580

[B14] Blake B. E. Pinney S. M. Hines E. P. Fenton S. E. Ferguson K. K. (2018). Associations between longitudinal serum perfluoroalkyl substance (PFAS) levels and measures of thyroid hormone, kidney function, and body mass index in the Fernald Community Cohort. Environ. Pollut. 242 (Pt A), 894–904. 10.1016/j.envpol.2018.07.042 30373035 PMC6309414

[B15] Chai J. Cai S. Y. Jiang Z. Wang H. Li Q. (2012). *Elevated hepatic multidrug resistance-associated protein 3/ATP-binding cassette subfamily C 3 expression in human obstructive cholestasis is mediated through tumor necrosis factor alpha and c-Jun NH* _2_ *-terminal kinase/stress-activated protein kinase-signaling pathway* . Hepatology 55 (5), 1485–1494. 10.1002/hep.24801 22105759 PMC3297707

[B16] Chiang J. Y. L. Ferrell J. M. (2020). Up to date on cholesterol 7 alpha-hydroxylase (CYP7A1) in bile acid synthesis. Liver Res. 4 (2), 47–63. 10.1016/j.livres.2020.05.001 34290896 PMC8291349

[B17] Chin C. F. Galam D. L. Gao L. Tan B. C. Wong B. H. Chua G. L. (2023). Blood-derived lysophospholipid sustains hepatic phospholipids and fat storage necessary for hepatoprotection in overnutrition. J. Clin. Investig. 133 (17), e171267. 10.1172/JCI171267 37463052 PMC10471173

[B18] Choi Y. H. Lee J. Y. Moon K. W. (2023). Exposure to volatile organic compounds and polycyclic aromatic hydrocarbons is associated with the risk of non-alcoholic fatty liver disease in Korean adolescents: Korea National Environmental Health Survey (KoNEHS) 2015-2017. Ecotoxicol. Environ. Saf. 251, 114508. 10.1016/j.ecoenv.2023.114508 36621033

[B19] Costello E. Rock S. Stratakis N. Eckel S. P. Walker D. I. Valvi D. (2022). Exposure to per- and polyfluoroalkyl substances and markers of liver injury: a systematic review and meta-analysis. Environ. Health Perspect. 130 (4), 46001. 10.1289/EHP10092 35475652 PMC9044977

[B20] D’Adamo R. Pelosi S. Trotta P. Sansone G. (1997). Bioaccumulation and biomagnification of polycyclic aromatic hydrocarbons in aquatic organisms. Mar. Chem. 56, 45–49. 10.1016/s0304-4203(96)00042-4

[B21] Das K. P. Wood C. R. Lin M. T. Starkov A. A. Lau C. Wallace K. B. (2017). Perfluoroalkyl acids-induced liver steatosis: effects on genes controlling lipid homeostasis. Toxicology 378, 37–52. 10.1016/j.tox.2016.12.007 28049043 PMC5994610

[B22] de la Garza R. G. Morales-Garza L. A. Martin-Estal I. Castilla-Cortazar I. (2017). Insulin-like growth factor-1 deficiency and cirrhosis establishment. J. Clin. Med. Res. 9 (4), 233–247. 10.14740/jocmr2761w 28270882 PMC5330765

[B23] Elcombe C. R. Elcombe B. M. Foster J. R. Chang S. C. Ehresman D. J. Noker P. E. (2012). Evaluation of hepatic and thyroid responses in male Sprague Dawley rats for up to eighty-four days following seven days of dietary exposure to potassium perfluorooctanesulfonate. Toxicology 293 (1-3), 30–40. 10.1016/j.tox.2011.12.015 22239858

[B24] Eriksen K. T. Raaschou-Nielsen O. McLaughlin J. K. Lipworth L. Tjønneland A. Overvad K. (2013). Association between plasma PFOA and PFOS levels and total cholesterol in a middle-aged Danish population. PLoS One 8 (2), e56969. 10.1371/journal.pone.0056969 23441227 PMC3575486

[B25] Fenton S. E. Ducatman A. Boobis A. DeWitt J. C. Lau C. Ng C. (2021). Per- and polyfluoroalkyl substance toxicity and human health review: current state of knowledge and strategies for informing future research. Environ. Toxicol. Chem. 40 (3), 606–630. 10.1002/etc.4890 33017053 PMC7906952

[B26] Fu L. Dong S. S. Xie Y. W. Tai L. S. Chen L. Kong K. L. (2010). Down-regulation of tyrosine aminotransferase at a frequently deleted region 16q22 contributes to the pathogenesis of hepatocellular carcinoma. Hepatology 51 (5), 1624–1634. 10.1002/hep.23540 20209601

[B27] Gallo V. Leonardi G. Genser B. Lopez-Espinosa M. J. Frisbee S. J. Karlsson L. (2012). Serum perfluorooctanoate (PFOA) and perfluorooctane sulfonate (PFOS) concentrations and liver function biomarkers in a population with elevated PFOA exposure. Environ. Health Perspect. 120 (5), 655–660. 10.1289/ehp.1104436 22289616 PMC3346788

[B28] Gwinn W. M. Auerbach S. S. Parham F. Stout M. D. Waidyanatha S. Mutlu E. (2020). Evaluation of 5-day *in vivo* rat liver and kidney with high-throughput transcriptomics for estimating benchmark doses of apical outcomes. Toxicol. Sci. 176 (2), 343–354. 10.1093/toxsci/kfaa081 32492150 PMC7416315

[B29] Hammarstrand S. Jakobsson K. Andersson E. Xu Y. Li Y. Olovsson M. (2021). Perfluoroalkyl substances (PFAS) in drinking water and risk for polycystic ovarian syndrome, uterine leiomyoma, and endometriosis: a Swedish cohort study. Environ. Int. 157, 106819. 10.1016/j.envint.2021.106819 34391986

[B30] Han H. Cho J. W. Lee S. Yun A. Kim H. Bae D. (2018). TRRUST v2: an expanded reference database of human and mouse transcriptional regulatory interactions. Nucleic Acids Res. 46 (D1), D380-D386–D386. 10.1093/nar/gkx1013 29087512 PMC5753191

[B31] Harris C. R. Millman K. J. van der Walt S. J. Gommers R. Virtanen P. Cournapeau D. (2020). Array programming with NumPy. Nature 585 (7825), 357–362. 10.1038/s41586-020-2649-2 32939066 PMC7759461

[B32] Haug M. Dunder L. Lind P. M. Lind L. Salihovic S. (2023). Associations of perfluoroalkyl substances (PFAS) with lipid and lipoprotein profiles. J. Expo. Sci. Environ. Epidemiol. 33 (5), 757–765. 10.1038/s41370-023-00545-x 37019983 PMC10541331

[B33] He A. (2020). Acetyl-CoA derived from hepatic peroxisomal beta-oxidation inhibits autophagy and promotes steatosis via mTORC1 activation. Mol. Cell. 79 (1), 30–42.e34. 10.1016/j.molcel.2020.05.007 32473093 PMC7335356

[B34] Heintz M. M. Chappell G. A. Thompson C. M. Haws L. C. (2022). Evaluation of transcriptomic responses in livers of mice exposed to the short-chain PFAS compound HFPO-DA. Front. Toxicol. 4, 937168. 10.3389/ftox.2022.937168 35832492 PMC9271854

[B35] Huang D. W. Sherman B. T. Stephens R. Baseler M. W. Lane H. C. Lempicki R. A. (2008). DAVID gene ID conversion tool. Bioinformation 2 (10), 428–430. 10.6026/97320630002428 18841237 PMC2561161

[B36] Hunter J. D. (2007). Matplotlib: a 2D graphics environment. Comput. Sci Eng 9, 90–95. 10.1109/mcse.2007.55

[B37] Igarashi Y. Nakatsu N. Yamashita T. Ono A. Ohno Y. Urushidani T. (2015). Open TG-GATEs: a large-scale toxicogenomics database. Nucleic Acids Res. 43, D921–D927. 10.1093/nar/gku955 25313160 PMC4384023

[B38] Jiang Z. Y. Zhou Y. Zhou L. Li S. W. Wang B. M. (2021). Identification of key genes and immune infiltrate in nonalcoholic steatohepatitis: a bioinformatic analysis. Biomed. Res. Int. 2021, 7561645. 10.1155/2021/7561645 34552988 PMC8452393

[B39] Jolliffe I. T. (1982). A note on the use of principal components in regression. J. R. Stat. Soc. Ser. C. Appl. Stat. 31 (3), 300–303. 10.2307/2348005

[B40] Kanehisa M. Goto S. (2000). KEGG: Kyoto Encyclopedia of genes and Genomes. Nucleic Acids Res. 28 (1), 27–30. 10.1093/nar/28.1.27 10592173 PMC102409

[B41] Khalil N. Chen A. Lee M. Czerwinski S. A. Ebert J. R. DeWitt J. C. (2016). Association of perfluoroalkyl substances, bone mineral density, and osteoporosis in the U.S. population in NHANES 2009-2010. Environ. Health Perspect. 124 (1), 81–87. 10.1289/ehp.1307909 26058082 PMC4710590

[B42] Kim H. S. Jun Kwack S. Sik Han E. Seok Kang T. Hee Kim S. Young Han S. (2011). Induction of apoptosis and CYP4A1 expression in Sprague-Dawley rats exposed to low doses of perfluorooctane sulfonate. J. Toxicol. Sci. 36 (2), 201–210. 10.2131/jts.36.201 21467747

[B43] Langmead B. Trapnell C. Pop M. Salzberg S. L. (2009). Ultrafast and memory-efficient alignment of short DNA sequences to the human genome. Genome Biol. 10 (3), R25. 10.1186/gb-2009-10-3-r25 19261174 PMC2690996

[B44] Li L. O. Ellis J. M. Paich H. A. Wang S. Gong N. Altshuller G. (2009). Liver-specific loss of long chain acyl-CoA synthetase-1 decreases triacylglycerol synthesis and beta-oxidation and alters phospholipid fatty acid composition. J. Biol. Chem. 284 (41), 27816–27826. 10.1074/jbc.M109.022467 19648649 PMC2788832

[B45] Li Y. Barregard L. Xu Y. Scott K. Pineda D. Lindh C. H. (2020). Associations between perfluoroalkyl substances and serum lipids in a Swedish adult population with contaminated drinking water. Environ. Health 19 (1), 33. 10.1186/s12940-020-00588-9 32169067 PMC7071576

[B46] Lichtenstein L. Berbée J. F. P. van Dijk S. J. van Dijk K. W. Bensadoun A. Kema I. P. (2007). Angptl4 upregulates cholesterol synthesis in liver via inhibition of LPL- and HL-dependent hepatic cholesterol uptake. Arterioscler. Thromb. Vasc. Biol. 27 (11), 2420–2427. 10.1161/ATVBAHA.107.151894 17761937

[B47] Liu B. Gao L. Ding L. Lv L. Yu Y. (2023). Trophodynamics and bioaccumulation of polycyclic aromatic hydrocarbons (PAHs) in marine food web from Laizhou Bay, China. Mar. Pollut. Bull. 194 (Pt B), 115307. 10.1016/j.marpolbul.2023.115307 37478788

[B48] Liu H. Pathak P. Boehme S. Chiang J. Y. L. (2016). Cholesterol 7α-hydroxylase protects the liver from inflammation and fibrosis by maintaining cholesterol homeostasis. J. Lipid Res. 57 (10), 1831–1844. 10.1194/jlr.M069807 27534992 PMC5036364

[B49] Louisse J. Fragki S. Rijkers D. Janssen A. van Dijk B. Leenders L. (2023). Determination of *in vitro* hepatotoxic potencies of a series of perfluoroalkyl substances (PFASs) based on gene expression changes in HepaRG liver cells. Arch. Toxicol. 97 (4), 1113–1131. 10.1007/s00204-023-03450-2 36864359 PMC10025204

[B50] Mallah M. A. Changxing L. Noreen S. Liu Y. Saeed M. (2022). Polycyclic aromatic hydrocarbon and its effects on human health: an overeview. Chemosphere 296, 133948. 10.1016/j.chemosphere.2022.133948 35151703

[B51] Mansouri K. Grulke C. M. Judson R. S. Williams A. J. (2018). OPERA models for predicting physicochemical properties and environmental fate endpoints. J. Cheminform 10 (1), 10. 10.1186/s13321-018-0263-1 29520515 PMC5843579

[B52] MathWorks Inc. (2022) MATLAB version: 9.12.0 (R2022a).

[B53] Mav D. Shah R. R. Howard B. E. Auerbach S. S. Bushel P. R. Collins J. B. (2018). A hybrid gene selection approach to create the S1500+ targeted gene sets for use in high-throughput transcriptomics. PLoS One 13 (2), e0191105. 10.1371/journal.pone.0191105 29462216 PMC5819766

[B54] Mellor C. L. Steinmetz F. P. Cronin M. T. (2016). The identification of nuclear receptors associated with hepatic steatosis to develop and extend adverse outcome pathways. Crit. Rev. Toxicol. 46 (2), 138–152. 10.3109/10408444.2015.1089471 26451809

[B55] Melzer D. Rice N. Depledge M. H. Henley W. E. Galloway T. S. (2010). Association between serum perfluorooctanoic acid (PFOA) and thyroid disease in the U.S. National Health and Nutrition Examination Survey. Environ. Health Perspect. 118 (5), 686–692. 10.1289/ehp.0901584 20089479 PMC2866686

[B56] Mortensen H. M. Senn J. Levey T. Langley P. Williams A. J. (2021). The 2021 update of the EPA's adverse outcome pathway database. Sci. Data 8 (1), 169. 10.1038/s41597-021-00962-3 34253739 PMC8275694

[B57] Natsoulis G. Pearson C. I. Gollub J. P Eynon B. Ferng J. Nair R. (2008). The liver pharmacological and xenobiotic gene response repertoire. Mol. Syst. Biol. 4, 175. 10.1038/msb.2008.9 18364709 PMC2290941

[B58] Nelson J. W. Hatch E. E. Webster T. F. (2010). Exposure to polyfluoroalkyl chemicals and cholesterol, body weight, and insulin resistance in the general U.S. population. Environ. Health Perspect. 118 (2), 197–202. 10.1289/ehp.0901165 20123614 PMC2831917

[B59] Pan J. Cen L. Zhou T. Yu M. Chen X. Jiang W. (2021). Insulin-like growth factor binding protein 1 ameliorates lipid accumulation and inflammation in nonalcoholic fatty liver disease. J. Gastroenterol. Hepatol. 36 (12), 3438–3447. 10.1111/jgh.15627 34273192

[B60] Patel A. B. Shaikh S. Jain K. R. Desai C. Madamwar D. (2020). Polycyclic aromatic hydrocarbons: sources, toxicity, and remediation approaches. Front. Microbiol. 11, 562813. 10.3389/fmicb.2020.562813 33224110 PMC7674206

[B61] Qiu Z. Qu K. Luan F. Liu Y. Zhu Y. Yuan Y. (2020). Binding specificities of estrogen receptor with perfluorinated compounds: a cross species comparison. Environ. Int. 134, 105284. 10.1016/j.envint.2019.105284 31707300

[B62] Rengarajan T. Rajendran P. Nandakumar N. Lokeshkumar B. Rajendran P. Nishigaki I. (2015). Exposure to polycyclic aromatic hydrocarbons with special focus on cancer. Asian Pac. J. Trop. Biomed. 5 (3), 182–189. 10.1016/s2221-1691(15)30003-4

[B63] Rosen M. B. Das K. P. Rooney J. Abbott B. Lau C. Corton J. C. (2017). PPARα-independent transcriptional targets of perfluoroalkyl acids revealed by transcript profiling. Toxicology 387, 95–107. 10.1016/j.tox.2017.05.013 28558994 PMC6129013

[B64] Ross D. Siegel D. (2021). The diverse functionality of NQO1 and its roles in redox control. Redox Biol. 41, 101950. 10.1016/j.redox.2021.101950 33774477 PMC8027776

[B65] Roth K. Yang Z. Agarwal M. Liu W. Peng Z. Long Z. (2021). Exposure to a mixture of legacy, alternative, and replacement per- and polyfluoroalkyl substances (PFAS) results in sex-dependent modulation of cholesterol metabolism and liver injury. Environ. Int. 157, 106843. 10.1016/j.envint.2021.106843 34479135 PMC8490327

[B66] Sang L. Ge Y. Liu F. Wei K. Shen X. Zhang Y. (2024). Association between per- and polyfluoroalkyl substances and sex hormone levels in males based on human studies. Ecotoxicol. Environ. Saf. 271, 115998. 10.1016/j.ecoenv.2024.115998 38262091

[B67] Schlezinger J. J. Puckett H. Oliver J. Nielsen G. Heiger-Bernays W. Webster T. F. (2020). Perfluorooctanoic acid activates multiple nuclear receptor pathways and skews expression of genes regulating cholesterol homeostasis in liver of humanized PPARα mice fed an American diet. Toxicol. Appl. Pharmacol. 405, 115204. 10.1016/j.taap.2020.115204 32822737 PMC7503133

[B68] Schultz A. A. Stanton N. Shelton B. Pomazal R. Lange M. A. Irving R. (2023). Biomonitoring of perfluoroalkyl and polyfluoroalkyl substances (PFAS) from the survey of the health of Wisconsin (SHOW) 2014-2016 and comparison with the national health and nutrition examination survey (NHANES). J. Expo. Sci. Environ. Epidemiol. 33 (5), 766–777. 10.1038/s41370-023-00593-3 37580384 PMC10804284

[B69] Sciome (2022). Sciome. GeniE. Available at: https://apps.sciome.com/genie/home (accessed on January 12, 2024).

[B70] Sen P. Qadri S. Luukkonen P. K. Ragnarsdottir O. McGlinchey A. Jäntti S. (2022). Exposure to environmental contaminants is associated with altered hepatic lipid metabolism in non-alcoholic fatty liver disease. J. Hepatol. 76 (2), 283–293. 10.1016/j.jhep.2021.09.039 34627976

[B71] Shannon P. Markiel A. Ozier O. Baliga N. S. Wang J. T. Ramage D. (2003). Cytoscape: a software environment for integrated models of biomolecular interaction networks. Genome Res. 13 (11), 2498–2504. 10.1101/gr.1239303 14597658 PMC403769

[B72] Society for Advancement of AOPs (2012). AOP-Wiki. Available at: http://aopwiki.org (accessed on April 12, 2024).

[B73] Steenland K. Tinker S. Frisbee S. Ducatman A. Vaccarino V. (2009). Association of perfluorooctanoic acid and perfluorooctane sulfonate with serum lipids among adults living near a chemical plant. Am. J. Epidemiol. 170 (10), 1268–1278. 10.1093/aje/kwp279 19846564

[B74] Takacs M. L. Abbott B. D. (2007). Activation of mouse and human peroxisome proliferator-activated receptors (alpha, beta/delta, gamma) by perfluorooctanoic acid and perfluorooctane sulfonate. Toxicol. Sci. 95 (1), 108–117. 10.1093/toxsci/kfl135 17047030

[B75] Tessari P. Coracina A. Cosma A. Tiengo A. (2009). Hepatic lipid metabolism and non-alcoholic fatty liver disease. Nutr. Metab. Cardiovasc Dis. 19 (4), 291–302. 10.1016/j.numecd.2008.12.015 19359149

[B76] The Pandas Development Team (2023) pandas-dev/pandas: pandas.

[B77] UniProt C. (2014). Orm1. Available at: https://www.uniprot.org/uniprotkb/P02764/entry (accessed on February 14, 2024).

[B78] UniProt C. (2022). Adh5. Available at: https://www.uniprot.org/uniprotkb/P12711/entry (accessed on February 15, 2024).

[B79] UniProt C. (2023a). UniProt: the universal protein knowledgebase in 2023. Nucleic Acids Res. 51 (D1), D523–D531. 10.1093/nar/gkac1052 36408920 PMC9825514

[B80] UniProt C. (2023b). Ehhadh. Available at: https://www.uniprot.org/uniprotkb/P07896/entry (accessed on February 14, 2024).

[B81] United States Environmental Protection Agency (2022). CompTox chemicals dashboard: 11H-Benzo[b]fluorene 243-17-4 | DTXSID1022477: executive summary 2021. Available at: https://comptox.epa.gov/dashboard/chemical/executive-summary/DTXSID1022477 (accessed on January 17, 2024).

[B82] United States Environmental Protection Agency (2023). CompTox chemicals dashboard: perfluorohexanesulfonamide 41997-13-1 | DRXSID5049320: executive summary 2021. Available at: https://comptox.epa.gov/dashboard/chemical/executive-summary/DTXSID50469320 (accessed on January 17, 2024).

[B83] United States Environmental Protection Agency (2024a). CompTox chemicals dashboard: 6:1 fluorotelomer alcohol 375-82-6 | DTXSID00190950: executive summary 2021. Available at: https://comptox.epa.gov/dashboard/chemical/executive-summary/DTXSID00190950, accessed on January 17, 2024

[B84] United States Environmental Protection Agency (2024b). Final PFAS national primary drinking water regulation. Available at: https://www.epa.gov/sdwa/and-polyfluoroalkyl-substances-pfas (accessed on April 22, 2024).

[B85] United States Environmental Protection Agency. PFAS national primary drinking water regulation rulemaking, march 29, 2023. Fed. Regist. 88 (6), 18638–18754.

[B86] Wan H. T. Zhao Y. G. Wei X. Hui K. Y. Giesy J. P. Wong C. K. C. (2012). PFOS-induced hepatic steatosis, the mechanistic actions on β-oxidation and lipid transport. Biochim. Biophys. Acta 1820 (7), 1092–1101. 10.1016/j.bbagen.2012.03.010 22484034

[B87] Wang H. Yan S. Cui J. Liang Y. Ren S. (2022). Adverse effects of perfluorooctane sulfonate on the liver and relevant mechanisms. Toxics 10 (5), 265. 10.3390/toxics10050265 35622678 PMC9144769

[B88] Wang Y. Zhang Y. Qian H. Lu J. Zhang Z. Min X. (2013). The g0/g1 switch gene 2 is an important regulator of hepatic triglyceride metabolism. PLoS One 8 (8), e72315. 10.1371/journal.pone.0072315 23951308 PMC3741160

[B89] Waskom M. (2021). Seaborn: statistical data visualization. Open Source Softw. 6 (60), 3021. 10.21105/joss.03021

[B90] Wolf C. J. Takacs M. L. Schmid J. E. Lau C. Abbott B. D. (2008). Activation of mouse and human peroxisome proliferator-activated receptor alpha by perfluoroalkyl acids of different functional groups and chain lengths. Toxicol. Sci. 106 (1), 162–171. 10.1093/toxsci/kfn166 18713766

[B91] Wu Y.-L. Ye J. Zhang S. Zhong J. Xi R. P. (2004). Clinical significance of serum IGF-I, IGF-II and IGFBP-3 in liver cirrhosis. World J. Gastroenterol. 10 (18), 2740–2743. 10.3748/wjg.v10.i18.2740 15309731 PMC4572205

[B92] Xie X. Weng X. Liu S. Chen J. Guo X. Gao X. (2021). Perfluoroalkyl and polyfluoroalkyl substance exposure and association with sex hormone concentrations: results from the NHANES 2015-2016. Environ. Sci. Eur. 33 (1), 69. 10.1186/s12302-021-00508-9 36061407 PMC9440377

[B93] Xu C. Liu Q. Liang J. Weng Z. Xu J. Jiang Z. (2021). Urinary biomarkers of polycyclic aromatic hydrocarbons and their associations with liver function in adolescents. Environ. Pollut. 278, 116842. 10.1016/j.envpol.2021.116842 33711626

[B94] Ye Q. Liu Y. Zhang G. Deng H. Wang X. Tuo L. (2023). Deficiency of gluconeogenic enzyme PCK1 promotes metabolic-associated fatty liver disease through PI3K/AKT/PDGF axis activation in male mice. Nat. Commun. 14 (1), 1402. 10.1038/s41467-023-37142-3 36918564 PMC10015095

[B95] Yeakley J. M. Shepard P. J. Goyena D. E. VanSteenhouse H. C. McComb J. D. Seligmann B. E. (2017). A trichostatin A expression signature identified by TempO-Seq targeted whole transcriptome profiling. PLoS One 12 (5), e0178302. 10.1371/journal.pone.0178302 28542535 PMC5444820

[B96] Yu L. Hammer R. E. Li-Hawkins J. Von Bergmann K. Lutjohann D. Cohen J. C. (2002). Disruption of Abcg5 and Abcg8 in mice reveals their crucial role in biliary cholesterol secretion. Proc. Natl. Acad. Sci. U. S. A. 99 (25), 16237–16242. 10.1073/pnas.252582399 12444248 PMC138595

[B97] Zhang X. Heckmann B. L. Campbell L. E. Liu J. (2017). G0S2: a small giant controller of lipolysis and adipose-liver fatty acid flux. Biochim. Biophys. Acta Mol. Cell. Biol. Lipids 1862 (10 Pt B), 1146–1154. 10.1016/j.bbalip.2017.06.007 28645852 PMC5890940

[B98] Zhang X. Zhao L. Ducatman A. Deng C. von Stackelberg K. E. Danford C. J. (2023). Association of per- and polyfluoroalkyl substance exposure with fatty liver disease risk in US adults. JHEP Rep. 5 (5), 100694. 10.1016/j.jhepr.2023.100694 36968216 PMC10033989

[B99] Zhou S. Guo C. Dai Y. Pan X. Luo X. Qin P. (2024). Association between polycyclic aromatic hydrocarbon exposure and liver function: the mediating roles of inflammation and oxidative stress. Environ. Pollut. 342, 123068. 10.1016/j.envpol.2023.123068 38042471

