# Supplementary figures and images for: Exposure to PFAS chemicals induces sex-dependent alterations in key rate-limiting steps of lipid metabolism in liver steatosis
Source: Front Toxicol. 2024 Jun 5;6:1390196. doi: 10.3389/ftox.2024.1390196 (PMC11188372; doi:10.3389/ftox.2024.1390196)

Expression of all target genes of MIEs

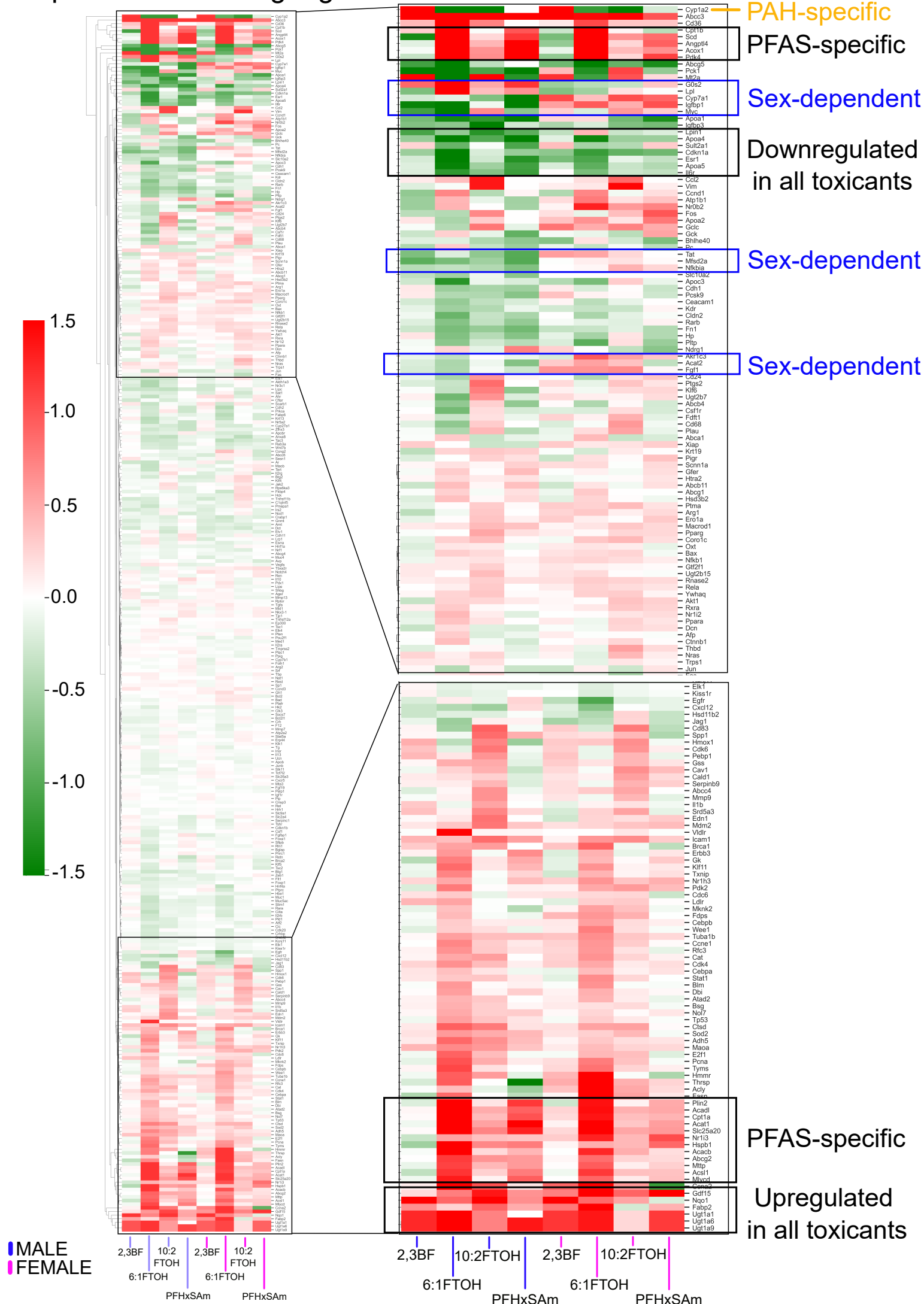

Supplement: Supplementary file 1 [file DataSheet2.pdf]
