# Supplementary material for: Exposure to PFAS chemicals induces sex-dependent alterations in key rate-limiting steps of lipid metabolism in liver steatosis
Source: Front Toxicol. 2024 Jun 5;6:1390196. doi: 10.3389/ftox.2024.1390196 (PMC11188372; doi:10.3389/ftox.2024.1390196)
Supplement: Supplementary file 2 [file Table1.docx]

Supplementary Material

# Supplementary Tables

**Supplementary Table S1**. Chemicals used as input to ToxProfiler to predict MIEs of PFAS and PAH chemicals

| **Compound class** | **Name** | **SMILES** |
| --- | --- | --- |
| PAH | 2,3-Benzofluorene* | C1C2=CC=CC=C2C3=CC4=CC=CC=C4C=C31 |
| PFAS | PFHxSAm* | C(C(C(C(F)(F)S(=O)(=O)N)(F)F)(F)F)(C(C(F)(F)F)(F)F)(F)F |
| PFAS | 6:1 FTOH* | FC(F)(F)C(F)(F)C(F)(F)C(F)(F)C(F)(F)C(F)(F)CO |
| PFAS | 10:2 FTOH* | FC(F)(F)C(F)(F)C(F)(F)C(F)(F)C(F)(F)C(F)(F)C(F)(F)C(F)(F)C(F)(F)C(F)(F)CCO |
| PFECA | HFPO-TA | C(=O)(C(C(F)(F)F)(OC(C(C(F)(F)F)(OC(C(C(F)(F)F)(F)F)(F)F)F)(F)F)F)F |
| PFECA | HFPO-DA | C(=O)(C(C(F)(F)F)(OC(C(C(F)(F)F)(F)F)(F)F)F)O |
| PFSA | PFDS | C(C(C(C(C(C(F)(F)S(=O)(=O)O)(F)F)(F)F)(F)F)(F)F)(C(C(C(C(F)(F)F)(F)F)(F)F)(F)F)(F)F |
| PFSA | PFOS | C(C(C(C(C(F)(F)S(=O)(=O)O)(F)F)(F)F)(F)F)(C(C(C(F)(F)F)(F)F)(F)F)(F)F |
| PFSA | PFHpS | C(C(C(C(F)(F)F)(F)F)(F)F)(C(C(C(F)(F)S(=O)(=O)O)(F)F)(F)F)(F)F |
| PFSA | PFHxS | C(C(C(C(F)(F)S(=O)(=O)O)(F)F)(F)F)(C(C(F)(F)F)(F)F)(F)F |
| PFSA | PFBS | C(C(C(F)(F)S(=O)(=O)O)(F)F)(C(F)(F)F)(F)F |
| PFCA | PFHxDA | C(=O)(C(C(C(C(C(C(C(C(C(C(C(C(C(C(C(F)(F)F)(F)F)(F)F)(F)F)(F)F)(F)F)(F)F)(F)F)(F)F)(F)F)(F)F)(F)F)(F)F)(F)F)(F)F)O |
| PFCA | PFTeDA | C(=O)(C(C(C(C(C(C(C(C(C(C(C(C(C(F)(F)F)(F)F)(F)F)(F)F)(F)F)(F)F)(F)F)(F)F)(F)F)(F)F)(F)F)(F)F)(F)F)O |
| PFCA | PFDoDA | C(=O)(C(C(C(C(C(C(C(C(C(C(C(F)(F)F)(F)F)(F)F)(F)F)(F)F)(F)F)(F)F)(F)F)(F)F)(F)F)(F)F)O |
| PFCA | PFUnDA | C(=O)(C(C(C(C(C(C(C(C(C(C(F)(F)F)(F)F)(F)F)(F)F)(F)F)(F)F)(F)F)(F)F)(F)F)(F)F)O |
| PFCA | PFDA | C(=O)(C(C(C(C(C(C(C(C(C(F)(F)F)(F)F)(F)F)(F)F)(F)F)(F)F)(F)F)(F)F)(F)F)O |
| PFCA | PFNA | C(=O)(C(C(C(C(C(C(C(C(F)(F)F)(F)F)(F)F)(F)F)(F)F)(F)F)(F)F)(F)F)O |
| PFCA | PFOA | C(=O)(C(C(C(C(C(C(C(F)(F)F)(F)F)(F)F)(F)F)(F)F)(F)F)(F)F)O |
| PFCA | PFHpA | C(=O)(C(C(C(C(C(C(F)(F)F)(F)F)(F)F)(F)F)(F)F)(F)F)O |
| PFCA | PFHxA | C(=O)(C(C(C(C(C(F)(F)F)(F)F)(F)F)(F)F)(F)F)O |
| PFCA | PFPeA | C(=O)(C(C(C(C(F)(F)F)(F)F)(F)F)(F)F)O |

An asterisk (*) marks the compounds analyzed in this study.
**MIEs**, molecular initiating events; **PAH**, polycyclic aromatic hydrocarbons; **PFAS**, per- or polyfluoro alkyl substances; **PFECA**, perfluoroalkyl ether carboxylic acids; **PFSA**, perfluoroalkyl sulfonic acids; **PFCA**, perfluoroalkyl carboxylic acids; **PFHxSAm**, perfluorohexanesulfonamide; **6:1 FTOH**, 6:1 fluorotelomer alcohol; **10:2 FTOH**, 10:2 fluorotelomer alcohol; **HFPO-TA**, hexafluoropropylene oxide trimer acid; **HFPO-DA**, hexafluoropropylene oxide dimer acid; **PFDS**, perfluorodecane sulfonate; **PFOS**, perfluorooctane sulfonate; **PFHpS**, perfluoroheptane sulfonate; **PFHxS**, perfluorohexane sulfonate; **PFBS**, perfluorobutane sulfonate; **PFHxDA**, perfluorohexadecanoic acid; **PFTeDA**, perfluorotetradecanoic acid; **PFDoDA**, perfluorododecanoic acid; **PFUnDA**, perfluoroundecanoic acid; **PFDA**, perfluorodecanoic acid; **PFNA**, perfluorononanoic acid; **PFOA**, perfluorooctanoic acid; **PFHpA**, perfluoroheptanoic acid; **PFHxA**, perfluorohexanoic acid; **PFPeA**, perfluoropentanoic acid.

**Supplementary Table S2**. Number of target genes up- (red cells) or downregulated (green cells) for each MIE in response to PFAS or PAH exposure. MIEs were obtained from ToxProfiler and published AOPs and their target genes were obtained from TRRUST database.

|  | **2,3-Benzofluorene** | | | | | | **6:1 FTOH** | | | | | | **10:2 FTOH** | | | | | | **PFHxSAm** | | | | | |
| --- | --- | --- | --- | --- | --- | --- | --- | --- | --- | --- | --- | --- | --- | --- | --- | --- | --- | --- | --- | --- | --- | --- | --- | --- |
|  | **Female** | | | **Male** | | | **Female** | | | **Male** | | | **Female** | | | **Male** | | | **Female** | | | **Male** | | |
| **Dose**  **(mg/kg)** | 111 | 333 | 1000 | 111 | 333 | 1000 | 111 | 333 | 1000 | 37 | 111 | 333 | 55 | 160 | 475 | 55 | 160 | 475 | 12 | 37 | 111 | 12 | 37 | 111 |
| AHR | 3 | 3 | 4 | 4 | 3 | 4 | 0 | 2 | 7 | 1 | 3 | 6 | 1 | 1 | 1 | 3 | 4 | 6 | 0 | 0 | 3 | 0 | 0 | 1 |
| AR | 0 | 0 | 0 | 0 | 1 | 0 | 0 | 0 | 3 | 1 | 2 | 2 | 1 | 2 | 3 | 0 | 2 | 7 | 0 | 0 | 0 | 0 | 0 | 2 |
| ESR1 | 0 | 1 | 2 | 1 | 1 | 1 | 0 | 2 | 7 | 2 | 4 | 9 | 2 | 2 | 0 | 1 | 5 | 9 | 0 | 0 | 2 | 1 | 0 | 2 |
| ESR2 | 0 | 0 | 0 | 0 | 0 | 0 | 0 | 0 | 0 | 0 | 0 | 1 | 0 | 0 | 0 | 0 | 1 | 2 | 0 | 0 | 1 | 0 | 0 | 0 |
| HNF4α | 0 | 0 | 0 | 0 | 0 | 0 | 0 | 0 | 0 | 0 | 0 | 0 | 0 | 0 | 0 | 0 | 0 | 1 | 0 | 0 | 0 | 0 | 0 | 0 |
| NFE2 | 0 | 1 | 1 | 0 | 0 | 0 | 0 | 0 | 0 | 0 | 0 | 0 | 0 | 0 | 1 | 0 | 0 | 1 | 0 | 0 | 0 | 0 | 0 | 0 |
| NR1H2 | 0 | 0 | 0 | 0 | 0 | 0 | 0 | 0 | 0 | 0 | 0 | 0 | 0 | 0 | 0 | 0 | 0 | 0 | 0 | 0 | 0 | 0 | 0 | 0 |
| NR1H3 | 0 | 0 | 0 | 0 | 0 | 0 | 0 | 0 | 0 | 0 | 0 | 0 | 0 | 0 | 0 | 0 | 0 | 0 | 0 | 0 | 0 | 0 | 0 | 0 |
| NR1H4 | 1 | 1 | 1 | 0 | 0 | 0 | 1 | 0 | 1 | 0 | 1 | 1 | 0 | 1 | 1 | 0 | 0 | 2 | 0 | 0 | 1 | 0 | 0 | 0 |
| NR1I2 | 1 | 1 | 1 | 1 | 2 | 1 | 0 | 2 | 2 | 0 | 2 | 2 | 0 | 1 | 1 | 1 | 1 | 3 | 0 | 0 | 1 | 0 | 0 | 0 |
| NR1I3 | 1 | 1 | 1 | 1 | 1 | 1 | 0 | 1 | 1 | 0 | 1 | 1 | 0 | 0 | 0 | 1 | 1 | 1 | 0 | 0 | 1 | 0 | 0 | 0 |
| NR3C1 | 1 | 1 | 1 | 1 | 1 | 1 | 0 | 1 | 5 | 0 | 2 | 4 | 0 | 0 | 1 | 1 | 1 | 4 | 0 | 0 | 1 | 0 | 0 | 0 |
| PPARα | 1 | 1 | 1 | 2 | 1 | 2 | 3 | 12 | 13 | 10 | 13 | 14 | 0 | 0 | 1 | 1 | 6 | 8 | 0 | 1 | 5 | 0 | 3 | 12 |
| PPARγ | 2 | 2 | 3 | 1 | 1 | 1 | 2 | 7 | 14 | 5 | 9 | 14 | 1 | 2 | 6 | 1 | 5 | 10 | 0 | 0 | 3 | 0 | 1 | 6 |
| RARα | 1 | 1 | 1 | 1 | 1 | 1 | 0 | 3 | 4 | 0 | 2 | 2 | 1 | 1 | 3 | 1 | 2 | 2 | 0 | 1 | 3 | 0 | 0 | 2 |
| RARβ | 0 | 0 | 0 | 0 | 0 | 0 | 0 | 0 | 0 | 0 | 1 | 1 | 0 | 0 | 0 | 0 | 0 | 1 | 0 | 0 | 0 | 0 | 0 | 0 |
| RARγ | 0 | 0 | 0 | 0 | 0 | 0 | 0 | 0 | 1 | 0 | 0 | 0 | 0 | 0 | 1 | 0 | 1 | 1 | 0 | 0 | 0 | 0 | 0 | 0 |
| RXRα | 2 | 2 | 2 | 1 | 1 | 1 | 1 | 2 | 2 | 0 | 2 | 2 | 1 | 2 | 2 | 1 | 1 | 1 | 0 | 0 | 2 | 0 | 0 | 2 |
| SREBF1 | 1 | 0 | 0 | 0 | 2 | 0 | 2 | 6 | 7 | 0 | 2 | 4 | 1 | 0 | 5 | 0 | 0 | 1 | 0 | 0 | 2 | 1 | 0 | 1 |
| AHR | 0 | 0 | 0 | 2 | 2 | 3 | 0 | 2 | 2 | 2 | 4 | 4 | 1 | 1 | 1 | 2 | 1 | 1 | 0 | 0 | 0 | 2 | 3 | 3 |
| AR | 0 | 2 | 1 | 2 | 2 | 2 | 0 | 1 | 2 | 1 | 1 | 4 | 2 | 3 | 2 | 2 | 1 | 2 | 0 | 0 | 3 | 1 | 2 | 3 |
| ESR1 | 0 | 2 | 2 | 1 | 1 | 2 | 1 | 1 | 4 | 1 | 2 | 3 | 1 | 1 | 1 | 0 | 0 | 0 | 0 | 0 | 2 | 1 | 2 | 3 |
| ESR2 | 0 | 1 | 1 | 0 | 0 | 0 | 0 | 1 | 1 | 0 | 1 | 2 | 1 | 1 | 1 | 0 | 0 | 0 | 0 | 0 | 1 | 0 | 1 | 1 |
| HNF4α | 0 | 1 | 2 | 2 | 1 | 3 | 1 | 2 | 3 | 4 | 8 | 7 | 2 | 4 | 3 | 0 | 1 | 2 | 0 | 2 | 3 | 2 | 4 | 7 |
| NFE2 | 0 | 0 | 0 | 0 | 0 | 0 | 0 | 0 | 0 | 0 | 0 | 0 | 0 | 0 | 0 | 0 | 0 | 0 | 0 | 0 | 0 | 0 | 0 | 0 |
| NR1H2 | 0 | 0 | 0 | 0 | 0 | 0 | 0 | 0 | 0 | 0 | 0 | 0 | 0 | 0 | 0 | 0 | 0 | 0 | 0 | 0 | 0 | 0 | 0 | 0 |
| NR1H3 | 0 | 0 | 1 | 0 | 0 | 0 | 0 | 0 | 0 | 0 | 0 | 0 | 0 | 0 | 0 | 0 | 0 | 0 | 0 | 1 | 1 | 0 | 0 | 1 |
| NR1H4 | 0 | 0 | 0 | 1 | 0 | 0 | 0 | 1 | 1 | 1 | 3 | 2 | 0 | 0 | 0 | 0 | 0 | 1 | 0 | 0 | 0 | 0 | 1 | 2 |
| NR1I2 | 0 | 0 | 0 | 0 | 0 | 0 | 0 | 0 | 0 | 0 | 1 | 1 | 0 | 0 | 0 | 0 | 0 | 0 | 0 | 0 | 0 | 0 | 0 | 3 |
| NR1I3 | 0 | 0 | 0 | 0 | 0 | 0 | 0 | 0 | 0 | 0 | 0 | 0 | 0 | 0 | 0 | 0 | 0 | 0 | 0 | 0 | 0 | 0 | 0 | 0 |
| NR3C1 | 0 | 1 | 1 | 1 | 1 | 2 | 0 | 1 | 1 | 0 | 1 | 2 | 1 | 1 | 1 | 1 | 0 | 1 | 0 | 0 | 1 | 1 | 1 | 3 |
| PPARα | 1 | 1 | 3 | 1 | 1 | 3 | 0 | 2 | 4 | 2 | 6 | 6 | 0 | 2 | 0 | 1 | 2 | 2 | 0 | 0 | 0 | 2 | 2 | 6 |
| PPARγ | 0 | 2 | 2 | 1 | 1 | 2 | 0 | 1 | 2 | 2 | 2 | 4 | 1 | 1 | 1 | 0 | 0 | 0 | 0 | 0 | 1 | 2 | 1 | 3 |
| RARα | 1 | 1 | 2 | 1 | 0 | 1 | 0 | 1 | 1 | 0 | 2 | 2 | 1 | 1 | 1 | 0 | 0 | 0 | 0 | 0 | 1 | 0 | 0 | 2 |
| RARβ | 0 | 0 | 0 | 0 | 0 | 0 | 0 | 0 | 0 | 0 | 1 | 1 | 0 | 0 | 0 | 0 | 0 | 0 | 0 | 0 | 0 | 0 | 0 | 1 |
| RARγ | 0 | 0 | 0 | 0 | 0 | 0 | 0 | 0 | 0 | 0 | 1 | 1 | 0 | 0 | 0 | 0 | 0 | 0 | 0 | 0 | 0 | 0 | 0 | 1 |
| RXRα | 0 | 0 | 0 | 1 | 0 | 0 | 0 | 0 | 0 | 0 | 1 | 0 | 0 | 0 | 0 | 0 | 0 | 1 | 0 | 0 | 0 | 0 | 1 | 1 |
| SREBF1 | 0 | 0 | 1 | 3 | 2 | 3 | 0 | 0 | 2 | 4 | 3 | 3 | 0 | 0 | 0 | 2 | 2 | 2 | 1 | 0 | 1 | 3 | 1 | 5 |

**MIEs**, molecular initiating events; **PAH**, polycyclic aromatic hydrocarbons; **PFAS**, per- or polyfluoro alkyl substances; **PFHxSAm**, perfluorohexanesulfonamide; **6:1 FTOH**, 6:1 fluorotelomer alcohol; **10:2 FTOH**, 10:2 fluorotelomer alcohol; **AHR**, aryl hydrocarbon receptor; **AR**, androgen receptor; **ESR1/2**, estrogen receptor 1/2; **HNF4α**, hepatocyte nuclear factor 4α; **NFE2**, nuclear factor erythroid 2; **NR1H2/3/4**, nuclear receptor subfamily 1, group H, member 2/3/4; **NR1I2**, nuclear receptor subfamily 1, group I, member 2; **NR3C1**, nuclear receptor subfamily 3, group C, member 1; **PPARα/γ**, peroxisome proliferator-activated receptor α/γ; **RAR**, retinoic acid receptor; **RXR**, retinoid X receptor; **SREBF1**, sterol regulatory element binding transcription factor 1.

**Supplementary Table S3**. Average alanine aminotransferase and aspartate aminotransferase levels in serum of rats exposed to the highest and lowest (control) concentrations of each toxicant at day 5 of exposure

| **Sex** | **Chemical** | **Dose,**  **mg/kg** | **ALT,**  **IU/L** | | | **AST,**  **IU/L** | | | **Cholesterol,**  **mg/dL** | | | **TG,**  **mg/dL** | | |
| --- | --- | --- | --- | --- | --- | --- | --- | --- | --- | --- | --- | --- | --- | --- |
| **Female** | **2,3-Benzofluorene** | **0** | 47.3 | ± | 2.9 | 69.90 | ± | 1.91 | 105.0 | ± | 5.3 | 49.0 | ± | 2.6 |
|  |  | **1,000** | 46.4 | ± | 6.3 | 71.40 | ± | 7.98 | 162.4 | ± | 5.8 | 64.0 | ± | 10.6 |
|  | **6:1 FTOH** | **0** | 50.8 | ± | 2.3 | 72.80 | ± | 1.78 | 95.6 | ± | 4.9 | 70.9 | ± | 10.4 |
|  |  | **1,000** | 62.0 | ± | 1.8 | 103.00 | ± | 19.79 | 54.0 | ± | 6.8 | 143.2 | ± | 25.2 |
|  | **10:2 FTOH** | **0** | 50.2 | ± | 2.8 | 78.50 | ± | 2.13 | 107.8 | ± | 3.4 | 57.6 | ± | 8.4 |
|  |  | **475** | 58.4 | ± | 3.7 | 82.60 | ± | 0.60 | 103.8 | ± | 4.6 | 56.4 | ± | 4.6 |
|  | **PFHxSAm** | **0** | 57.9 | ± | 2.3 | 82.20 | ± | 1.88 | 93.2 | ± | 2.3 | 51.5 | ± | 3.7 |
|  |  | **111** | 69.8 | ± | 6.0 | 69.60 | ± | 1.57 | 86.6 | ± | 5.5 | 84.4 | ± | 13.8 |
| **Male** | **2,3-Benzofluorene** | **0** | 60.8 | ± | 4.7 | 74.90 | ± | 2.54 | 114.9 | ± | 5.9 | 96.6 | ± | 12.1 |
|  |  | **1,000** | 52.4 | ± | 5.2 | 72.80 | ± | 6.51 | 163.6 | ± | 7.1 | 85.8 | ± | 9.0 |
|  | **6:1 FTOH** | **0** | 61.2 | ± | 3.6 | 80.10 | ± | 4.72 | 106.6 | ± | 3.8 | 99.9 | ± | 9.9 |
|  |  | **333** | 209.0 | ± | 67.2 | 195.00 | ± | 54.03 | 69.2 | ± | 4.9 | 58.6 | ± | 14.8 |
|  | **10:2 FTOH** | **0** | 61.8 | ± | 3.1 | 80.22 | ± | 3.53 | 118.1 | ± | 3.7 | 100.8 | ± | 1.4 |
|  |  | **475** | 96.0 | ± | 10.5 | 122.25 | ± | 11.46 | 89.2 | ± | 5.2 | 98.5 | ± | 9.7 |
|  | **PFHxSAm** | **0** | 64.8 | ± | 4.0 | 79.22 | ± | 3.28 | 103.3 | ± | 4.4 | 85.8 | ± | 11.2 |
|  |  | **111** | 78.0 | ± | 12.1 | 15.80 | ± | 5.54 | 79.8 | ± | 4.0 | 65.8 | ± | 13.1 |

**ALT**, alanine aminotransferase; **AST**, aspartate aminotransferase; **TG**, triglyceride; **6:1 FTOH**, 6:1 fluorotelomer alcohol; **10:2 FTOH**, 10:2 fluorotelomer alcohol; **PFHxSAm**, perfluorohexanesulfonamide.

# Supplementary Figures

**
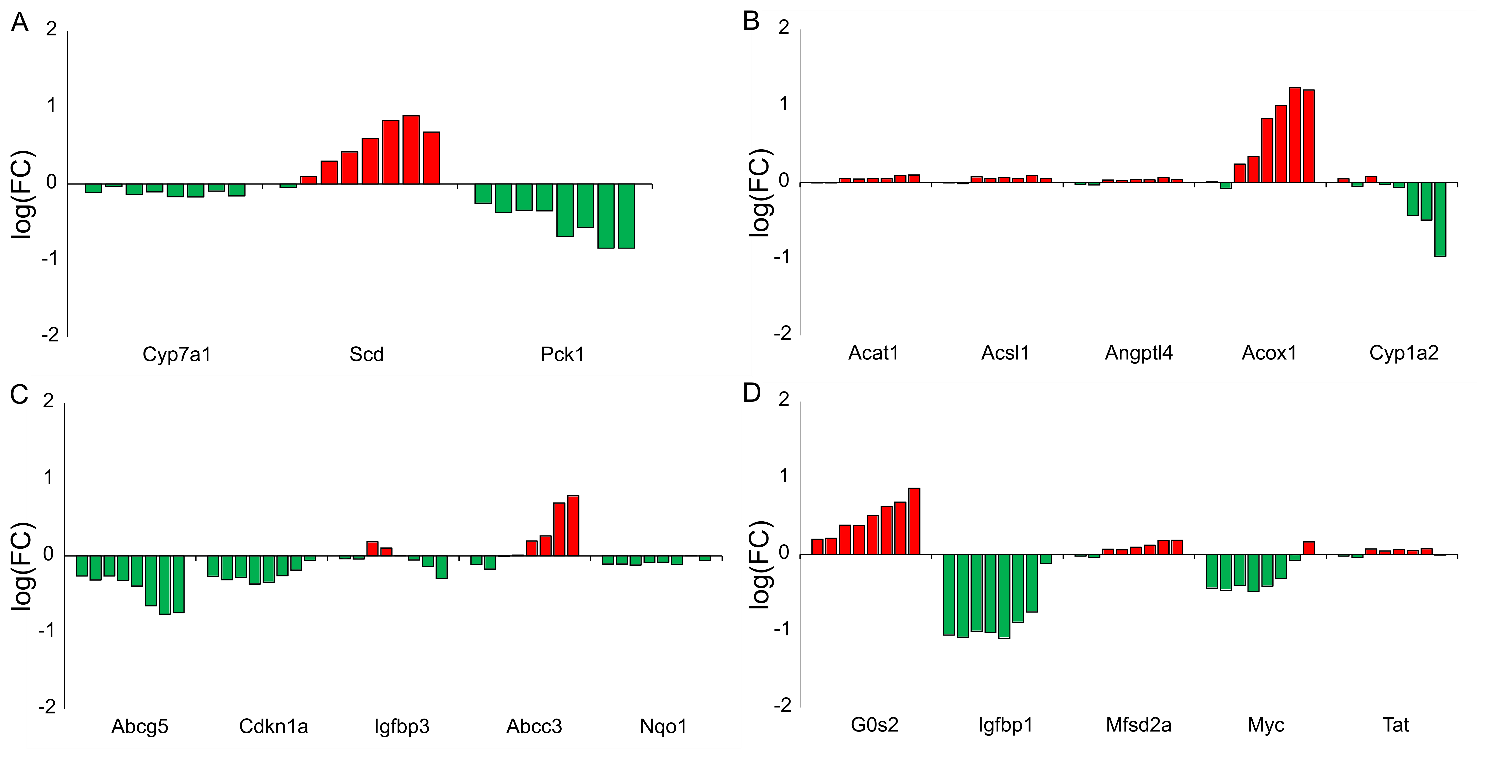
**

**Supplementary Figure S1**. Expression of key genes in response to perfluorooctanoic acid (PFOA) corroborates the findings of this study. The bar plots show the PFOA-induced fold-change (FC) values of the genes with (A) rate-limiting function, (B) per- or polyfluoro alkyl substances (PFAS)-specific and sex-independent responses, (C) similar responses to PFAS and non-PFAS compounds, and (D) sex-specific responses.


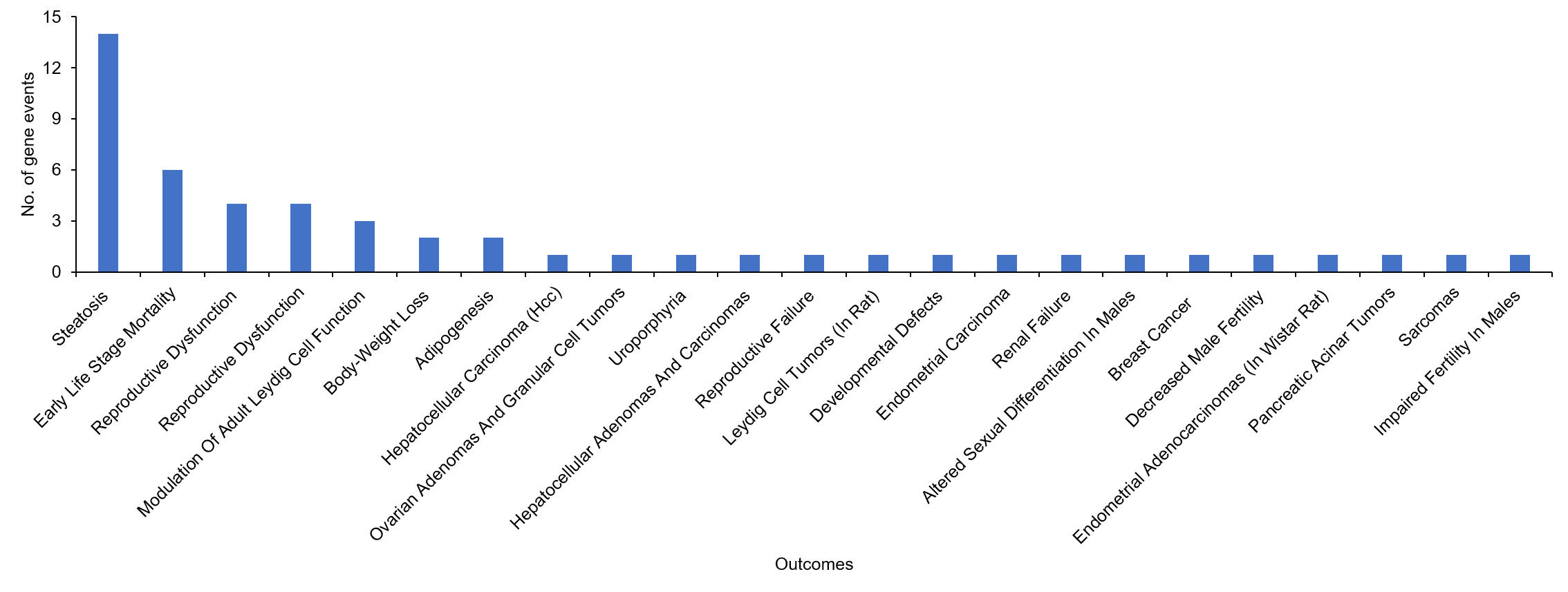
 **Supplementary Figure S2.** Alternate outcomes of target genes analyzed in this study, mined from the US environmental protection agency adverse outcome pathway database (EPA AOP-DB).
